# Supplementary material for: Improving Primary Care After Stroke (IPCAS) trial: protocol of a randomised controlled trial to evaluate a novel model of care for stroke survivors living in the community
Source: BMJ Open. 2019 Aug 18;9(8):e030285. doi: 10.1136/bmjopen-2019-030285 (PMC6701584; doi:10.1136/bmjopen-2019-030285)
Supplement: Supplementary data [file bmjopen-2019-030285supp001.pdf]

improving primary care after stroke

UNIVERSITY OF CAMBRIDGE
Public Health and Primary Care
The Primary Care Unit

University Hospitals of Leicester NHS
NHS Trust

Improving Primary Care After Stroke

TRIAL CONSENT FORM - STROKE SURVIVOR

Version 2.0

Participant ID: *<Participant ID>*

Please **initial** each box

1. I have read and understood the information sheet (*version, dated*) for the above study, and have had the opportunity to ask questions. ☐
2. I understand that I can stop taking part in the research at any time. If I stop I do not have to give a reason. My medical care or legal rights will not be affected. ☐
3. I understand that my GP will be informed of my participation in this study. ☐
4. I understand that the researchers may want to look at relevant sections of my medical notes. I give permission for researchers to have access to my medical records. ☐
5. I understand that my **confidential data** (e.g. my name and address) will be stored securely, and only accessed by the research team. ☐
6. I understand that my **anonymised** data will be archived and may be shared with other researchers and used in future studies. ☐
7. **I agree to take part in this study.** ☐

The following statement is **optional**. Please **only initial the box** if you **agree** to the statement.

8. I agree for my annual review to be audiotaped. ☐

Name of Participant: ________________________ **Date:** ________________

Signature: ______________________

Improving primary care after stroke (IPCAS): A cluster randomised controlled trial
Consent Form
IRAS Number: 233891 v 2.0 19-12-17
