# Supplementary material for: Improving Primary Care After Stroke (IPCAS) trial: protocol of a randomised controlled trial to evaluate a novel model of care for stroke survivors living in the community
Source: BMJ Open. 2019 Aug 18;9(8):e030285. doi: 10.1136/bmjopen-2019-030285 (PMC6701584; doi:10.1136/bmjopen-2019-030285)
Supplement: Author's manuscript [file bmjopen-2019-030285.draft_revisions.pdf]

BMJ Open

BMJ Open is committed to open peer review. As part of this commitment we make the peer review history of every article we publish publicly available.

When an article is published we post the peer reviewers' comments and the authors' responses online. We also post the versions of the paper that were used during peer review. These are the versions that the peer review comments apply to.

The versions of the paper that follow are the versions that were submitted during the peer review process. They are not the versions of record or the final published versions. They should not be cited or distributed as the published version of this manuscript.

BMJ Open is an open access journal and the full, final, typeset and author-corrected version of record of the manuscript is available on our site with no access controls, subscription charges or pay-per-view fees (http://bmjopen.bmj.com).

If you have any questions on BMJ Open's open peer review process please email info.bmjopen@bmj.com

The IPCAS Trial (Improving Primary Care After Stroke): protocol of a randomised controlled trial to evaluate a novel model of care for stroke survivors living in the community.

Journal:	*BMJ Open*
Manuscript ID	bmjopen-2019-030285
Article Type:	Protocol
Date Submitted by the Author:	12-Mar-2019

Complete List of Authors:	Mullis, Ricky; University of Cambridge, General Practice & Primary Care Research Centre<br>Aquino, Maria Raisa Jessica (Ryc); University of Cambridge, General Practice & Primary Care Research Centre<br>Dawson, Sarah; University of Cambridge Institute of Public Health, MRC Biostatistics Unit<br>Johnson, Vicki; Univ Hosp Leicester NHS Trust, Leicester Diabetees Centre<br>Jowett, Sue; University of Birmingham, Health Economics Unit<br>Kreit, Elizabeth; University of Cambridge, General Practice & Primary Care Research Centre<br>Mant, Jonathan; University of Cambridge, General Practice and Primary Care Research Unit

Keywords:	Stroke < NEUROLOGY, PRIMARY CARE, MLAS

SCHOLARONE™
Manuscripts

The IPCAS Trial (**I**mproving **P**rimary **C**are **A**fter **S**troke): protocol of a randomised controlled trial to evaluate a novel model of care for stroke survivors living in the community.

*Ricky Mullis[1], Maria Raisa Aquino[1], Sarah Dawson[2], Vicki Johnson[3], Sue Jowett[4], Elizabeth Kreit[1], Jonathan Mant[1].

On behalf of the IPCAS investigator team: Marian Carey, Melanie Davies, Yvonne Doherty, Kamlesh Khunti, Bundy Mackintosh, Adrian Mander, Christopher McKevitt, Martin Roland, Stephen Sutton, Marion Walker, Elizabeth Warburton.

1. Primary Care Unit, Department of Public Health and Primary Care, University of Cambridge, UK.
2. MRC Biostatistics Unit, Cambridge Institute of Public Health, University of Cambridge, UK.
3. Complex Intervention Team, Leicester Diabetes Centre, University Hospitals of Leicester NHS Trust, Leicester, UK.
4. Health Economics Unit, Institute of Applied Health Research, University of Birmingham, UK.

* Corresponding author r.mullis@medschl.cam.ac.uk

Key words: Stroke, primary care, MLAS.

Abstract

Introduction
Survival after stroke is improving, leading to increased demand on primary care and community services to meet the long-term care needs of people living with stroke. No formal primary care based model of care exists to support stroke survivors living in the community, and stroke survivors report that many of their needs are not being met. We have developed a multi-factorial primary care model to address these longer-term needs. We aim to evaluate the clinical and cost effectiveness of this new model of primary care for stroke survivors compared with standard care.

Methods and Analysis
IPCAS is a two-arm cluster-randomised controlled trial with general practices as the unit of randomisation. People on the stroke registers of GP practices in the East of England and East Midlands will be invited to participate. One arm will receive the IPCAS model of care including a structured review using a checklist; a self-management programme; enhanced communication pathways between primary care and specialist services; direct point of contact for patients. The other arm will receive usual care. We aim to recruit 920 people with history of stroke registered with 46 general practices.

The primary endpoint for the trial is two sub-scales (emotion and handicap) of the Stroke Impact Scale (SIS) as co-primary outcomes at 12 months (adjusted for baseline). Secondary outcomes include: SIS Short Form, EuroQol EQ-5D-5L, ICECAP-A, Southampton Stroke Self-management questionnaire (SSSQ), Health Literacy Questionnaire (HLQ); medication use. Cost-effectiveness of the new model will be determined in a within-trial economic evaluation.

Ethics and Dissemination
Favourable ethical opinion was gained from Yorkshire & The Humber-Bradford Leeds NHS Research Ethics Committee. Approval to start was given by the Health Research Authority (HRA) prior to recruitment of participants at any NHS site.

For peer review only - http://bmjopen.bmj.com/site/about/guidelines.xhtml

Patient and public involvement will help development of the dissemination plan.

Strengths and limitations of this study

Strengths: Systematically developed complex intervention
Randomised controlled design
Broad inclusion criteria
Patient reported outcomes
Clinical and economic evaluation

Limitations: Limited blinding of research team to treatment allocation
Exclusion of nursing home residents

Study Registration: ClinicalTrials.gov Identifier: NCT03353519 27th November 2017

Favourable ethical opinion for the research was gained on 19th December 2017 from Yorkshire & The Humber - Bradford Leeds NHS Research Ethics Committee. Approval to start was given by the Health Research Authority (HRA) on 21st December 2017, prior to the recruitment of participants commencing at any NHS site.

Patient recruitment started March 2018.

IRAS project ID: 233891 Protocol number: RG71908 REC reference: 17/YH/0441

The IPCAS trial is co-sponsored by the University of Cambridge and NHS Cambridgeshire & Peterborough Clinical Commissioning Group. This research is covered under Cambridge University's Public Liability and Professional Indemnity policy.

This study is funded by the National Institute for Health Research's Programme Grant for Applied Research titled 'Developing primary care services for stroke survivors' reference PTC-RP-PG-0213-20001. The views expressed are those of the authors and not necessarily those of the NHS, the NIHR or the Department of Health. The chief investigator for the study is Professor Jonathan Mant, University of Cambridge email:jm677@medschl.cam.ac.uk

Background and Rationale

Survival after stroke is improving[1,2] leading to increased demand on primary care services to meet the long-term care needs of people with stroke living in the community. Surveys suggest these needs are not being adequately addressed and that many stroke survivors are dissatisfied with care after discharge from hospital.[3,4] Approximately a third of stroke survivors have moderate to severe levels of disability at 6-months.[5] In addition to the many physical consequences of stroke, commonly reported areas of concern include information needs, feelings of abandonment, problems with communication[3] emotional, psychological and social problems, fatigue, and cognitive sequelae including poor memory and concentration.[6]

Little evidence exists as to how best to support long-term stroke survivors[7] especially beyond the first year after stroke,[8] and recent trials of greater specialist input post discharge from hospital have had mixed results.[7,9]

Primary care could play an important role in the care of people with stroke, including secondary prevention and risk factor management, supporting access to community services, facilitating transfer back to specialist services, and education and provision of

For peer review only - http://bmjopen.bmj.com/site/about/guidelines.xhtml

information about stroke. However, the feeling of ‘abandonment’ of people with stroke after hospital discharge suggests this role is not being fulfilled. Indeed, current recommendations,[10] such as for a structured review of needs beyond the first six weeks after discharge, are not being implemented.[11]

We have developed a novel multi-factorial primary care model to address the longer-term needs of stroke survivors living in the community.

Aims

The IPCAS trial (**I**mproving **P**rimary **C**are **A**fter **S**troke) aims to evaluate the clinical and cost effectiveness of a new model of primary care for stroke survivors living in the community compared with standard care.

The primary endpoint for the trial will be two sub-scales (emotion and handicap) of the Stroke Impact Scale (SIS v3.0)[12] as co-primary outcomes at 12 months (adjusted for baseline).

Trial Design

Two-arm cluster randomised controlled trial with general practices as the unit of randomisation.

Randomisation will be performed as random permuted block randomisation with a 1:1 allocation stratified by GP practice size.

Setting and participants

GP practices with a stroke register comprising a minimum of 100 patients and representing a range of urban/rural and different socio-economic status from the East of England and the East Midlands will be recruited. We aim to recruit approximately 920 people with a confirmed history of stroke registered with 46 general practices.

Inclusion Criteria

- On practice register with a history of stroke
- Able to provide written informed consent (with or without the help of a carer)
- Age 18 years or older

Exclusion Criteria

- Patients on the palliative care register
- Living in a nursing home

Methods

Recruitment of stroke survivors
Prior to practice level randomisation (see below) electronic searches of the clinical computer system will generate a list of people with a history of stroke who meet the inclusion criteria for the study.

For peer review only

For peer review only - http://bmjopen.bmj.com/site/about/guidelines.xhtml

Potentially eligible participants will be sent an invitation by their General Practice to take part in the study. The invitation pack contains an invitation cover letter, the Patient Information Sheet (PIS), consent form, a questionnaire containing the co-primary outcomes, and instructions to return the consent form and questionnaire to the researchers in a pre-paid envelope (provided). If no response is received within 2 weeks from the initial mail-out, the practice will send a reminder. If no response is received after the reminder then no further attempts at contact will be made.

Randomisation

Once all invitation letters and reminders have been sent out to patients within a practice, the GP practice will be randomised to intervention or control (ratio of 1:1). Randomisation will be performed centrally by the trial statistician using a stratified, random permuted block design. The stratification factor will be GP practice size, split into two levels: ≤10,500 and >10,500 patients, which reflects the median GP list size in the catchment area.

Figure 1 about here

Intervention

The new model of care incorporates a multi-faceted package of service aimed at providing a structured review of stroke care needs, a self-management programme for survivors and their carers, optimised communication between patients and health care services, enhanced communication pathways between the different care services, and increased awareness of and access to national and local community and charity provided services.

i) Structured Review of Patient Needs:

A structured review will be performed by a practice nurse or other appropriately trained member of the Practice team. Consenting patients will be invited for review by the practice in same the way that they would normally be contacted (e.g. post, telephone or SMS). Where practicable, this review will be incorporated into the regular annual review recommended by current guidelines.[10] A 15-item checklist of common post-stroke needs[13] adapted from a checklist recommended by the WSO[14] will be sent to the stroke survivor in advance, who will be asked to tick all needs which apply to them, and to bring this to the appointment. At the review, the patient will be asked which of the ticked items is their priority for immediate attention. Practice staff will discuss and address up to three key needs prioritised by the patient.

The review will last approximately 20-30 minutes and may include a routine physical check-up (e.g. blood pressure, record of immunisation, and medication review dependent upon normal clinical practice at the GP surgery) followed by the discussion of post-stroke care needs as identified by the stroke survivor. The outcome of the review will be an action plan agreed with the stroke survivor on how to address each of the key needs identified in the review.

The patient will be provided with an information leaflet introducing the self-management programme, with instructions on how to get further information and how to access the programme.

ii) Self-management Programme (MLAS):

"My Life After Stroke" (MLAS) is a theory-driven self-management education programme with an explicit philosophical underpinning for stroke survivors and their carers (where appropriate) consisting of an initial individual preparatory session, 4 weekly group-based sessions, and a final individual session. Individual appointments last approximately 30-45

For peer review only - http://bmjopen.bmj.com/site/about/guidelines.xhtml
For peer review only

minutes. Group sessions will include stroke survivors and their carers (where relevant) and lasts approximately 2½ hours (including breaks).

Group sessions cover a variety of topics including risk factors for stroke and prevention, psychological well-being, information, social needs, problem solving and goal setting. Participants will be given a handbook containing educational content and further information based on the session topics.

The programme will be run by two trained facilitators (health care professionals or people working in the voluntary sector) with an interest in or experience of stroke. All sessions will be held at a suitable, accessible, local community facility.

iii) Direct Point of Contact:
A direct point of contact at the GP surgery will be provided for stroke survivors and their carers. The staff member conducting the enhanced annual review will explain how to access the direct point of contact. Survivors or carers will be able to call the practice and indicate that they would like to talk to someone about a stroke related problem. A single or several Practice nurses or other appropriately trained health care members of the Practice team will assume the role. If none of these people are available at the time of the call, a designated member of the care team will phone back. The aim of the direct point of contact will be to provide support and advice for stroke specific issues, arranging follow-up appointments, and signposting to further specialist or community services.

iv) Enhanced Communication Pathways:
We will arrange a meeting between primary care staff from several practices and specialist staff (hospital and community) to facilitate primary/secondary care communication going forward. All Practice staff involved with the care of stroke survivors will be encouraged to attend additional training/ meetings organised by the specialist services, and given direct contact details for informal communication. Video recordings of local specialist(s) describing their service, the type of patients normally referred to the service, and ways of contacting the service will be made available to all general practice staff.

v) Service Mapping:
To support the information needs regarding local services for stroke related problems, the care team will be provided with a catalogue of stroke (and other relevant) services in participating localities, including information on how to access them. This resource will be available in several electronic and hard copy formats to enable easy access by staff at the practice.

vi) Training for General Practice Staff:
Training for practice staff involved in structured stroke reviews will include an overview of stroke and stroke related long-term needs, followed by discussion of vignettes based on items from the stroke review checklist. Practice staff will suggest and discuss with the research team the most suitable course of action in each situation tailored to local context.

The list of key health and social services available in the local area will be provided, and practice staff will be familiarised with the service mapping resource that will be made available to them at the practice. The outcomes of the structured review will be recorded on a template in the patient records. We will discuss with the practice how best to embed the direct point of contact role within the current practice operations. To enable ease of attendance the training will be held in the practice and will last approximately two hours.

For peer review only - http://bmjopen.bmj.com/site/about/guidelines.xhtml

Control Arm
General practices randomised to the control arm of the trial will continue to deliver usual stroke care.

Demographic data: age, gender, ethnicity and post code will be collected via postal questionnaire at the time of invitation to the study.

Primary Outcome

The primary endpoint for the trial will be two sub-scales (emotion and handicap) of the Stroke Impact Scale (SIS v3.0)[12] as co-primary outcomes at 12 months (adjusted for baseline) after randomisation of the Practice.

Secondary Outcomes
To be collected at baseline, six and 12 months:

- SIS Short Form[12]
- EuroQol EQ-5D-5L[15]
- ICEpop CAPability measure for Adults (ICECAP-A)[16]
- Time since stroke
- * Co-morbidity, medication use (prescription & "over the counter" (OTC))
- * Southampton Stroke Self-management questionnaire (SSSQ)[17]
- * Health Literacy Questionnaire (HLQ)[18]

* Collected at 12 months follow-up only.

Data Collection
In this pragmatic, practice level cluster-randomised trial blinding to treatment allocation of the research team or clinical staff involved in delivering the intervention or control condition is not possible. The primary outcome will be captured by postal questionnaires sent to participants. Only in the event of missing data from the primary outcome will participants be contacted by the research team to either encourage them to return their questionnaire or to complete missing items via telephone. Questionnaire data entry onto an electronic spreadsheet will be outsourced to a third-party provider via secure data transfer for blinded data entry. The "coded-allocation" spreadsheet will then be returned to the trial statistician, who will undertake all analyses independent of the rest of the research team.

Baseline: The primary outcome data (emotion and handicap sub-scales of the SIS) will be collected via postal questionnaire at the time of invitation to the study prior to randomisation of the practice. Secondary outcome data (SIS Short Form, EuroQol EQ-5D-5L, and ICEpop CAPability measure for Adults (ICECAP-A)) will be collected by postal questionnaire after receipt of consent. Non-responders to the secondary outcome questionnaire will be followed-up by telephone or the most appropriate method for a participant with aphasia.

Follow-up: at six and 12 months by postal questionnaire. Non-responders/incomplete responders will be followed up by telephone or the most appropriate method for a participant with aphasia.

A review of the general practice notes of consenting participants will be conducted. Data extracted will include number and nature of primary care visits, secondary care inpatient and outpatient visits, investigations, medications and use of social services.

For peer review only - http://bmjopen.bmj.com/site/about/guidelines.xhtml

Figure 2 about here.

Patient involvement

Patient and members of the public were involved at several stages of the trial, including the design, management, and conduct of the trial. We received input from stroke survivors in the design of the trial materials and management oversight through membership of the trial steering committee. We carefully assessed the burden of the trial interventions on patients. We will seek patient and public involvement in the development of an appropriate method of dissemination.

Statistical Methods and Analysis

Sample size

With 23 clusters per arm and an average of 20 patients per cluster, assuming an intra-class correlation of 0.03, a typical coefficient of variation of the cluster size of 0.65[19], and 2.5% significance (adjusted to 2.5% because of the use of two co-primary outcomes), we would be able to detect an effect size of 0.33 with at least 90% power on the co-primary outcomes (emotion and handicap sub-scales of the Stroke Impact Scale (SIS v3.0[12])). The sample size calculation has been inflated to allow for a rate of 20% loss to follow-up for patients within clusters. Loss to follow-up of entire clusters is not anticipated.

Analysis of Primary Outcome

We will use intention to treat (ITT) methods for the analysis of the primary end-points. A mixed effects model will be used to model each of the co-primary outcomes with a cluster random effect and fixed effects for the intervention and covariates that might potentially confound the relationship. Distributional assumptions will be assessed graphically by residual q-q plots and residual by fitted value plots. To handle the co-primary outcomes, 97.5% confidence intervals will be reported for the two primary treatment effects which are equivalent to having the Bonferonni correction on the planned 5% significance level for a single endpoint.

Missing data will be analysed under the assumptions of missing completely at random and missing at random. Multiple imputation will be used to impute missing outcome data and the various potential predictors of missingness will be included in the imputation model.

Economic evaluation

The cost-effectiveness (cost-utility) of the new system of care (intervention package) compared with usual care will be determined in a within-trial economic evaluation. Data will be collected via electronic primary care records and patient questionnaires on resource use implications of the intervention (including training), primary care visits, secondary care inpatient and outpatient visits, investigations, medications and use of social services. Patient and carer-incurred costs will also be considered to allow analysis from a broader societal perspective. Data collection will be undertaken within the trial to determine the time taken to deliver the structured review, and any additional resources required. Attendance at the individual and group MLAS sessions will also be recorded for every participant, and each session will be costed, taking into account staff time, any consumables and use of the venue. Standard unit costs will be applied to health care resource use including NHS reference costs, the BNF for medications and Unit Costs for Health and Social Care (PSSRU).

The main outcomes of interest from the trial are quality of life (measured using EQ 5D-5L[15] at baseline and six and 12 months after entry into the trial) and capability (using the ICECAP-A questionnaire[16]). Initially, a cost-consequence analysis will be performed, to present a disaggregated analysis of all mean resource use and costs related to the intervention and

For peer review only - http://bmjopen.bmj.com/site/about/guidelines.xhtml

usual care, health care, social care, patient/carer costs and EQ-5D-5L and ICECAP-A scores at all time points. Quality-adjusted life years (QALYs) will be calculated by the area-under-the-curve method using responses at all time points, and adjusted for baseline covariates including EQ-5D-5L score. Multiple imputation will be undertaken where there is missing cost and outcome data. An incremental cost-utility analysis will then be undertaken to determine the cost per QALY gained of the intervention compared with usual care.

To explore uncertainties in the analyses, deterministic sensitivity analysis is proposed to test the robustness of the results when varying key assumptions (for example, length of time required to deliver the intervention).

Process evaluation

A process evaluation will examine the implementation of the IPCAS trial using both quantitative and qualitative methods. As well as capturing process variables, the evaluation will also entail a multidimensional approach to assessing intervention fidelity – the extent to which an intervention is delivered as planned.[20] Using the US National Institutes of Health Behaviour Change Consortium (NIHBCC) guidance[21] we will conduct a 'whole picture' assessment of the intervention across five fidelity dimensions: 1) design, 2) training, 3) delivery, 4) receipt, and 5) enactment. An overview is provided below, with the full protocol reported elsewhere (currently in submission).

Fidelity of design will be assessed through mapping intervention components to its purported theoretical frameworks. All intervention components have been specified a priori and recorded. Additionally, treatment differentiation (i.e. extent to which intervention and control group practices differ) is considered by comparing the contents of the intervention vs usual care. Fidelity of training will be assessed using self-complete questionnaires (MLAS), video-recorded observations (MLAS) and audio-recorded observations (IPCAS). Fidelity of delivery will be assessed through audio-recorded observations (IPCAS), structured telephone calls to healthcare professionals, and direct observations (MLAS). In addition, semistructured interviews will be conducted with healthcare professionals delivering the intervention, which will help to assess both training and delivery. Fidelity of receipt and enactment will be assessed using self-complete questionnaires (MLAS), structured telephone calls and semistructured interviews with participants.

Analysis

Quantitative aspects of the process evaluation (e.g. process variables, coded video-recorded observations, self-complete questionnaires) will be synthesised descriptively. Qualitative aspects of the process evaluation (e.g. semistructured interviews, qualitative data from questionnaires) will be synthesised using deductive thematic analysis, using the specific domains from the NIHBCC guidance.

Reporting Adverse Events

Each Principal Investigator is responsible for reporting all non-exempt SAEs to the Chief Investigator (CI) within 24 hours of first notification. The CI is responsible for ensuring the assessment of all SAEs for expectedness and relatedness is completed and the onward notification of all non-exempt SAEs to the Sponsor within 24 hours of first notification.

Author Statement

All authors contributed to the design of the trial and drafting of the manuscript. The IPCAS investigator team supported the design of the trial and contributed to the writing of the final manuscript.

Trial Management

For peer review only - http://bmjopen.bmj.com/site/about/guidelines.xhtml

The trial is co-sponsored by NHS Cambridgeshire and Peterborough CCG and the University of Cambridge. The study team work with local Clinical Research Networks (CRNs) in the East of England and the East Midlands to identify and recruit GP practices.

Oversight of the trial will fall to an independent committee fulfilling the combined roles of Trial Steering Committee (TSC) and Data Monitoring Committee (DMC). They will provide overall supervision of the conduct of the trial on behalf of the trial sponsor(s) in accordance with NIHR recommendations.[22,23] There are no pre-specified criteria for electively stopping the trial prematurely. In the event that the joint TSC/DMC raise concerns over the safety of participants or the scientific integrity of the trial, a decision as to whether to continue will be discussed and voted upon in keeping with the Terms of Reference of the committees and with Good Clinical Practice in Research guidelines.

Data Management and storage

Data completed by participants, such as consent forms and questionnaires, will be returned to the study team via post using pre-paid stamped addressed envelopes. All relevant data collected at practice sites will be sent to the study team by trained and delegated practice staff via a secure transfer server. Paper data will be stored in locked filing cabinets within a security card-protected building at the University of Cambridge. Electronic data (including audio-recordings) will be stored on a Secure Data Hosting Service (SDHS) protected by a dual authentication located on a firewall-protected virtual network (VLAN). Access to study data is restricted to the study team by dual authentication and group permissions. All investigators and trial site staff involved in this trial will comply with the requirements of the General Data Protection Regulation (EU) 2016/679 with regards to the collection, storage, processing and disclosure of personal information.

Declaration of interests: None

References

1. Gulliford MC, Charlton J, Rudd A, et al. Declining 1-year case-fatality of stroke and increasing coverage of vascular risk management: population-based cohort study. Journal of Neurology, Neurosurgery & Psychiatry 2010;81(4):416-22.

2. Lee S, Shafe ACE, Cowie MR. UK stroke incidence, mortality and cardiovascular risk management 1999–2008: time-trend analysis from the General Practice Research Database. BMJ Open 2011;1(2).

3. McKevitt C, Fudge N, Redfern J, et al. Self-reported long-term needs after stroke. Stroke 2011;42(5):1398-403.

4. National Audit Office. Progress in improving stroke care. Department of Health, 2010.

5. Royal College of Physicians CEaEUobotISWP. Sentinel stroke national audit programme (SSNAP): clinical audit April - June 2014. Sentinel Stroke National Audit Programme (SSNAP); 2014.

6. Murray J, Young J, Forster A. Review of longer-term problems after a disabling stroke. Rev Clin Gerontol 2007;17(04):277-92.

For peer review only - http://bmjopen.bmj.com/site/about/guidelines.xhtml

7. Logan P, Armstrong S, Avery A, Barer D, Barton G, Darby J, et al. Rehabilitation aimed at improving outdoor mobility for people after stroke: a multi-centre randomised controlled study (the Getting out of the House Study). Health Technol Assess 2014;18(29)

8. Aziz Noor A, Leonardi-Bee J, Phillips Margaret F, Gladman J, Legg Lynn A, Walker M. Therapy-based rehabilitation services for patients living at home more than one year after stroke. Cochrane Database Syst Rev. 2008;(2): [CD005952 p.].

9. Rodgers H, Shaw L, Bhattarai N, Cant R, Drummond A, Ford G, Forster A, Francis R, Hills K, Howel D, Laverty A, McKevitt C, McMeekin P, Price C, Stamp E, Stevens E, Vale L. A trial to evaluate an extended rehabilitation service for stroke patients (EXTRAS): main results. European Stroke Journal 2018; 3(1S): 3–586 DOI: 10.1177/2396987318770127

10. Intercollegiate Stroke Working Party. National clinical guideline for stroke. London Royal College of Physicians. 2016.

11. Department of Health. National Stroke Strategy. London. Report Number: 284536, 2007.

12. Jenkinson, C., Fitzpatrick, R., Crocker, H. and Peters, M. (2013) The Stroke Impact Scale. Validation in a UK setting and development of a SIS short form and SIS index. Stroke 44 pp 2532-2535

13. Turner GM, Mullis R, Lim L, Kreit E, Mant J. Using a checklist to facilitate management of long-term care needs after stroke: Insights from focus groups and a feasibility study. BMC Family Practice *BMC Family Practice*; 20:2 https://doi.org/10.1186/s12875-018-0894-3

14. Philp I, Brainin M, Walker MF, et al. Development of a poststroke checklist to standardize follow-up care for stroke survivors. *J Stroke Cerebrovasc Dis*. 2013;22(7):e173–80

15. van Reenen M, Janssen B. EQ-5D-5L user guide: basic information on how to use the EQ-5D-5L instrument. Version 2.1. Rotterdam; 2015.

16. Al-Janabi H, N Flynn T, Coast J. Development of a self-report measure of capability wellbeing for adults: the ICECAP-A. Quality of Life Research. 2012; 21(1):167-76.

17. Boger, E., Hankins, M., Demain, S., and Latter, S. (2015) Development and psychometric evaluation of a new patient-reprted outcome measure for stroke self-management: The Southampton Stroke Self-Management Questionnaire (SSSMQ). Health and Quality of Life Outcomes; 13; 165

18. Osborne RH, Batterham R, Elsworth GR, Hawkins M, Buchbinder R. The grounded theory, psychometric development and initial validation of the Health Literacy Questionnaire (HLQ). BMC Public Health. 2013;13:658.

19. Campbell, M.J. and Walters, S.J. 2014. How to Design, Analyse and Report Cluster Randomised Trials in Medicine and Health Related Research. John Wiley & Sons. New York.

20. Peters DH, Adam T, Alonge O, Agyepong IA, Tran N. Republished research: Implementation research: what it is and how to do it. Br J Sports Med 2014;48:731-736.

For peer review only - http://bmjopen.bmj.com/site/about/guidelines.xhtml

21. Bellg, A. J., Borrelli, B., Resnick, B., Hecht, J., Minicucci, D. S., Ory, M., et al. Treatment Fidelity Workgroup of the NIH Behavior Change Consortium. (2004). Enhancing Treatment Fidelity in Health Behavior Change Studies: Best Practices and Recommendations From the NIH Behavior Change Consortium. Health Psychology, 23(5), 443-451. http://dx.doi.org/10.1037/0278-6133.23.5.443

22. NIHR Good Clinical Practice (GCP) Reference Guide (Version 3.1 August 2016) https://www.nihr.ac.uk/our-faculty/documents/GCPReferenceGuide.pdf

23. NIHR: A Guide to Efficient Trial Management; Trial-Managers'-Network-Guide Fourth Edition: January 2014 ISBN 9780854329748

For peer review only

For peer review only - http://bmjopen.bmj.com/site/about/guidelines.xhtml

Figure 1. **Flow Chart of Study**

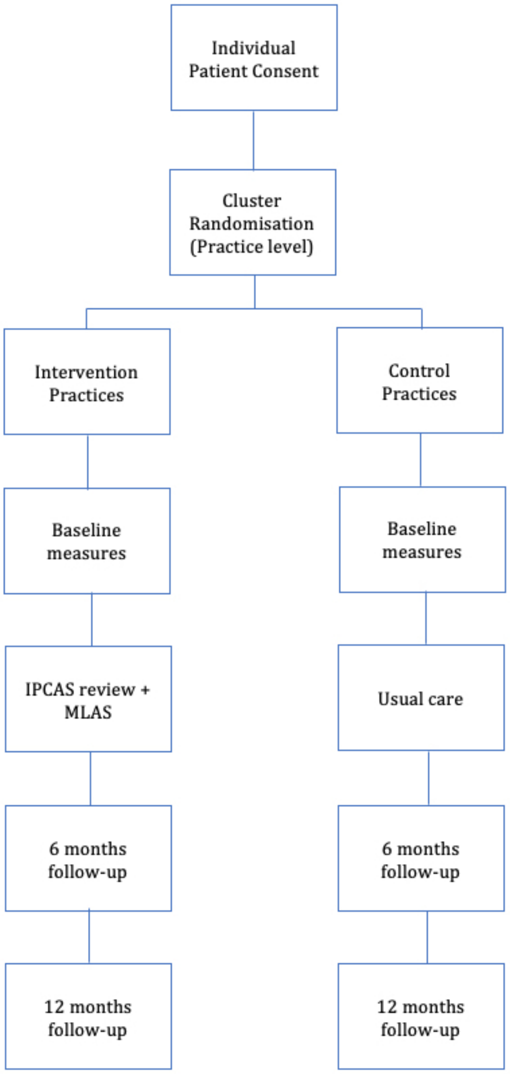

IPCAS = Improving Primary Care After Stroke
MLAS = My Life After Stroke

Figure 1. IPCAS Flowchart of Trial

201x313mm (72 x 72 DPI)

Figure 2. Scheduled enrolment, interventions, and assessments of participants.

	STUDY PERIOD			
	**Enrolment**	**Allocation**	**Post-allocation**	
**TIMEPOINT**	**$-t_1$**	**0**	***6 months***	***12 months***
**ENROLMENT:**				
**Informed consent**	X			
**Allocation**		X		
**INTERVENTIONS:**				
[Intervention]		←	—	→
[Control]		←	—	→
**ASSESSMENTS:**				
[Baseline variables]	X			
[Primary outcomes]			X	X
[Secondary outcomes]			X	X

Figure 2 IPCAS SPIRIT flowchart

208x168mm (72 x 72 DPI)

STANDARD PROTOCOL ITEMS: RECOMMENDATIONS FOR INTERVENTIONAL TRIALS

SPIRIT 2013 Checklist: Recommended items to address in a clinical trial protocol and related documents*

Section/item	Item No	Description	Addressed on page number
**Administrative information**			
Title	1	Descriptive title identifying the study design, population, interventions, and, if applicable, trial acronym	_____1_______
Trial registration	2a	Trial identifier and registry name. If not yet registered, name of intended registry	_____2_______
	2b	All items from the World Health Organization Trial Registration Data Set	____1 - 6______
Protocol version	3	Date and version identifier	___Footer_____
Funding	4	Sources and types of financial, material, and other support	_____2_______
Roles and responsibilities	5a	Names, affiliations, and roles of protocol contributors	_____1_______
	5b	Name and contact information for the trial sponsor	_____2_______
	5c	Role of study sponsor and funders, if any, in study design; collection, management, analysis, and interpretation of data; writing of the report; and the decision to submit the report for publication, including whether they will have ultimate authority over any of these activities	_____2_______
	5d	Composition, roles, and responsibilities of the coordinating centre, steering committee, endpoint adjudication committee, data management team, and other individuals or groups overseeing the trial, if applicable (see Item 21a for data monitoring committee)	_____9______

For peer review only

Introduction

Background and rationale	6a	Description of research question and justification for undertaking the trial, including summary of relevant studies (published and unpublished) examining benefits and harms for each intervention	2
	6b	Explanation for choice of comparators	2
Objectives	7	Specific objectives or hypotheses	3
Trial design	8	Description of trial design including type of trial (eg, parallel group, crossover, factorial, single group), allocation ratio, and framework (eg, superiority, equivalence, noninferiority, exploratory)	3

Methods: Participants, interventions, and outcomes

Study setting	9	Description of study settings (eg, community clinic, academic hospital) and list of countries where data will be collected. Reference to where list of study sites can be obtained	3
Eligibility criteria	10	Inclusion and exclusion criteria for participants. If applicable, eligibility criteria for study centres and individuals who will perform the interventions (eg, surgeons, psychotherapists)	3
Interventions	11a	Interventions for each group with sufficient detail to allow replication, including how and when they will be administered	4 – 6
	11b	Criteria for discontinuing or modifying allocated interventions for a given trial participant (eg, drug dose change in response to harms, participant request, or improving/worsening disease)	N/A
	11c	Strategies to improve adherence to intervention protocols, and any procedures for monitoring adherence (eg, drug tablet return, laboratory tests)	4
	11d	Relevant concomitant care and interventions that are permitted or prohibited during the trial	N/A
Outcomes	12	Primary, secondary, and other outcomes, including the specific measurement variable (eg, systolic blood pressure), analysis metric (eg, change from baseline, final value, time to event), method of aggregation (eg, median, proportion), and time point for each outcome. Explanation of the clinical relevance of chosen efficacy and harm outcomes is strongly recommended	6
Participant timeline	13	Time schedule of enrolment, interventions (including any run-ins and washouts), assessments, and visits for participants. A schematic diagram is highly recommended (see Figure)	Figure 2

Sample size	14	Estimated number of participants needed to achieve study objectives and how it was determined, including clinical and statistical assumptions supporting any sample size calculations	_____7_______
Recruitment	15	Strategies for achieving adequate participant enrolment to reach target sample size	______________
**Methods: Assignment of interventions (for controlled trials)**			
Allocation:			
Sequence generation	16a	Method of generating the allocation sequence (eg, computer-generated random numbers), and list of any factors for stratification. To reduce predictability of a random sequence, details of any planned restriction (eg, blocking) should be provided in a separate document that is unavailable to those who enrol participants or assign interventions	_____4_______
Allocation concealment mechanism	16b	Mechanism of implementing the allocation sequence (eg, central telephone; sequentially numbered, opaque, sealed envelopes), describing any steps to conceal the sequence until interventions are assigned	_____4_______
Implementation	16c	Who will generate the allocation sequence, who will enrol participants, and who will assign participants to interventions	_____4_______
Blinding (masking)	17a	Who will be blinded after assignment to interventions (eg, trial participants, care providers, outcome assessors, data analysts), and how	_____6_______
	17b	If blinded, circumstances under which unblinding is permissible, and procedure for revealing a participant's allocated intervention during the trial	_____6_______
**Methods: Data collection, management, and analysis**			
Data collection methods	18a	Plans for assessment and collection of outcome, baseline, and other trial data, including any related processes to promote data quality (eg, duplicate measurements, training of assessors) and a description of study instruments (eg, questionnaires, laboratory tests) along with their reliability and validity, if known. Reference to where data collection forms can be found, if not in the protocol	_____6_____
	18b	Plans to promote participant retention and complete follow-up, including list of any outcome data to be collected for participants who discontinue or deviate from intervention protocols	_____6_______

For peer review only - http://bmjopen.bmj.com/site/about/guidelines.xhtml

Data management	19	Plans for data entry, coding, security, and storage, including any related processes to promote data quality (eg, double data entry; range checks for data values). Reference to where details of data management procedures can be found, if not in the protocol	______9______
Statistical methods	20a	Statistical methods for analysing primary and secondary outcomes. Reference to where other details of the statistical analysis plan can be found, if not in the protocol	_____7 - 8____
	20b	Methods for any additional analyses (eg, subgroup and adjusted analyses)	______7______
	20c	Definition of analysis population relating to protocol non-adherence (eg, as randomised analysis), and any statistical methods to handle missing data (eg, multiple imputation)	______7______
**Methods: Monitoring**			
Data monitoring	21a	Composition of data monitoring committee (DMC); summary of its role and reporting structure; statement of whether it is independent from the sponsor and competing interests; and reference to where further details about its charter can be found, if not in the protocol. Alternatively, an explanation of why a DMC is not needed	______9______
	21b	Description of any interim analyses and stopping guidelines, including who will have access to these interim results and make the final decision to terminate the trial	______9_____
Harms	22	Plans for collecting, assessing, reporting, and managing solicited and spontaneously reported adverse events and other unintended effects of trial interventions or trial conduct	______8______
Auditing	23	Frequency and procedures for auditing trial conduct, if any, and whether the process will be independent from investigators and the sponsor	______9______
**Ethics and dissemination**			
Research ethics approval	24	Plans for seeking research ethics committee/institutional review board (REC/IRB) approval	______2______
Protocol amendments	25	Plans for communicating important protocol modifications (eg, changes to eligibility criteria, outcomes, analyses) to relevant parties (eg, investigators, REC/IRBs, trial participants, trial registries, journals, regulators)	_____________

Consent or assent	26a	Who will obtain informed consent or assent from potential trial participants or authorised surrogates, and how (see Item 32)	______3______
	26b	Additional consent provisions for collection and use of participant data and biological specimens in ancillary studies, if applicable	_____N/A_____
Confidentiality	27	How personal information about potential and enrolled participants will be collected, shared, and maintained in order to protect confidentiality before, during, and after the trial	______9______
Declaration of interests	28	Financial and other competing interests for principal investigators for the overall trial and each study site	______9______
Access to data	29	Statement of who will have access to the final trial dataset, and disclosure of contractual agreements that limit such access for investigators	______9______
Ancillary and post-trial care	30	Provisions, if any, for ancillary and post-trial care, and for compensation to those who suffer harm from trial participation	______2______
Dissemination policy	31a	Plans for investigators and sponsor to communicate trial results to participants, healthcare professionals, the public, and other relevant groups (eg, via publication, reporting in results databases, or other data sharing arrangements), including any publication restrictions	______7______
	31b	Authorship eligibility guidelines and any intended use of professional writers	_____N/A_____
	31c	Plans, if any, for granting public access to the full protocol, participant-level dataset, and statistical code	______________
**Appendices**			
Informed consent materials	32	Model consent form and other related documentation given to participants and authorised surrogates	______________
Biological specimens	33	Plans for collection, laboratory evaluation, and storage of biological specimens for genetic or molecular analysis in the current trial and for future use in ancillary studies, if applicable	_____N/A_____

*It is strongly recommended that this checklist be read in conjunction with the SPIRIT 2013 Explanation & Elaboration for important clarification on the items. Amendments to the protocol should be tracked and dated. The SPIRIT checklist is copyrighted by the SPIRIT Group under the Creative Commons "Attribution-NonCommercial-NoDerivs 3.0 Unported" license.

For peer review only - http://bmjopen.bmj.com/site/about/guidelines.xhtml

BMJ Open

The IPCAS Trial (Improving Primary Care After Stroke): protocol of a randomised controlled trial to evaluate a novel model of care for stroke survivors living in the community.

Journal:	*BMJ Open*
Manuscript ID	bmjopen-2019-030285.R1
Article Type:	Protocol
Date Submitted by the Author:	21-May-2019
Complete List of Authors:	Mullis, Ricky; University of Cambridge, General Practice & Primary Care Research Centre<br>Aquino, Maria Raisa Jessica (Ryc); University of Cambridge, General Practice & Primary Care Research Centre<br>Dawson, Sarah; University of Cambridge Institute of Public Health, MRC Biostatistics Unit<br>Johnson, Vicki; Univ Hosp Leicester NHS Trust, Leicester Diabetees Centre<br>Jowett, Sue; University of Birmingham, Health Economics Unit<br>Kreit, Elizabeth; University of Cambridge, General Practice & Primary Care Research Centre<br>Mant, Jonathan; University of Cambridge, General Practice and Primary Care Research Unit
**Primary Subject Heading**:	Cardiovascular medicine
Secondary Subject Heading:	Neurology
Keywords:	Stroke < NEUROLOGY, PRIMARY CARE, MLAS, IPCAS, self management

SCHOLARONE™
Manuscripts

The IPCAS Trial (**I**mproving **P**rimary **C**are **A**fter **S**troke): protocol of a randomised controlled trial to evaluate a novel model of care for stroke survivors living in the community.

*Ricky Mullis[1], Maria Raisa Aquino[1], Sarah Dawson[2], Vicki Johnson[3], Sue Jowett[4], Elizabeth Kreit[1], Jonathan Mant[1].

On behalf of the IPCAS investigator team: Marian Carey, Melanie Davies, Yvonne Doherty, Kamlesh Khunti, Lisa Lim, Bundy Mackintosh, Adrian Mander, Christopher McKevitt, Martin Roland, Stephen Sutton, Marion Walker, Elizabeth Warburton.

1. Primary Care Unit, Department of Public Health and Primary Care, University of Cambridge, UK.
2. MRC Biostatistics Unit, Cambridge Institute of Public Health, University of Cambridge, UK.
3. Complex Intervention Team, Leicester Diabetes Centre, University Hospitals of Leicester NHS Trust, Leicester, UK.
4. Health Economics Unit, Institute of Applied Health Research, University of Birmingham, UK.

* Corresponding author r.mullis@medschl.cam.ac.uk

Key words: Stroke, primary care, self management, IPCAS, MLAS.

Abstract

Introduction
Survival after stroke is improving, leading to increased demand on primary care and community services to meet the long-term care needs of people living with stroke. No formal primary care based holistic model of care with clinical trial evidence exists to support stroke survivors living in the community, and stroke survivors report that many of their needs are not being met. We have developed a multi-factorial primary care model to address these longer-term needs. We aim to evaluate the clinical and cost effectiveness of this new model of primary care for stroke survivors compared with standard care.

Methods and Analysis
IPCAS is a two-arm cluster-randomised controlled trial with general practices as the unit of randomisation. People on the stroke registers of GP practices will be invited to participate. One arm will receive the IPCAS model of care including a structured review using a checklist; a self-management programme; enhanced communication pathways between primary care and specialist services; direct point of contact for patients. The other arm will receive usual care. We aim to recruit 920 people with stroke registered with 46 general practices.

The primary endpoint is two sub-scales (emotion and handicap) of the Stroke Impact Scale (SIS) as co-primary outcomes at 12 months (adjusted for baseline). Secondary outcomes include: SIS Short Form, EuroQol EQ-5D-5L, ICECAP-A, Southampton Stroke Self-management questionnaire (SSSQ), Health Literacy Questionnaire (HLQ); medication use. Cost-effectiveness of the new model will be determined in a within-trial economic evaluation.

Ethics and Dissemination
Favourable ethical opinion was gained from Yorkshire & Humber-Bradford Leeds NHS Research Ethics Committee. Approval to start was given by the Health Research Authority (HRA) prior to recruitment of participants at any NHS site. Data will be presented at national and international conferences and published in peer-reviewed journals.

For peer review only - http://bmjopen.bmj.com/site/about/guidelines.xhtml

Patient and public involvement helped develop the dissemination plan.

Strengths and limitations of this study

Strengths: Systematically developed complex intervention
Randomised controlled design
Broad inclusion criteria

Limitations: Limited blinding of the research team to treatment allocation
Exclusion of nursing home residents

Study Registration: ClinicalTrials.gov Identifier: NCT03353519 27th November 2017

Favourable ethical opinion for the research was gained on 19th December 2017 from Yorkshire & The Humber - Bradford Leeds NHS Research Ethics Committee. Approval to start was given by the Health Research Authority (HRA) on 21st December 2017, prior to the recruitment of participants commencing at any NHS site.

Patient recruitment started March 2018.

IRAS project ID: 233891 Protocol number: RG71908 REC reference: 17/YH/0441

The IPCAS trial is co-sponsored by the University of Cambridge and NHS Cambridgeshire & Peterborough Clinical Commissioning Group. This research is covered under Cambridge University's Public Liability and Professional Indemnity policy.

This study is funded by the National Institute for Health Research's Programme Grant for Applied Research titled 'Developing primary care services for stroke survivors' reference PTC-RP-PG-0213-20001. The views expressed are those of the authors and not necessarily those of the NHS, the NIHR or the Department of Health. The chief investigator for the study is Professor Jonathan Mant, University of Cambridge email:jm677@medschl.cam.ac.uk

Background and Rationale

Survival after stroke is improving[1,2] leading to increased demand on primary care services to meet the long-term care needs of people with stroke living in the community. Surveys suggest these needs are not being adequately addressed and that many stroke survivors are dissatisfied with care after discharge from hospital.[3,4] Approximately a third of stroke survivors have moderate to severe levels of disability at 6-months.[5] In addition to the many physical consequences of stroke, commonly reported areas of concern include information needs, feelings of abandonment, problems with communication[3] emotional, psychological and social problems, fatigue, and cognitive sequelae including poor memory and concentration.[6]

Little evidence exists as to how best to support long-term stroke survivors[7] especially beyond the first year after stroke,[8] and recent trials of greater specialist input post discharge from hospital have had mixed results.[7,9] No formal primary care based holistic model of care with clinical trial evidence exists to support stroke survivors living in the community, and stroke survivors report that many of their needs are not being met. Systematic reviews have demonstrated that self-management after stroke shows promise, but evidence on aspects such as mood and social tasks remain sparse, with wide confidence intervals around effects on outcomes such as quality of life.[10-12]

For peer review only - http://bmjopen.bmj.com/site/about/guidelines.xhtml

Primary care could play an important role in the care of people with stroke, including secondary prevention and risk factor management, supporting access to community services, facilitating transfer back to specialist services, and education and provision of information about stroke. However, the feeling of 'abandonment' of people with stroke after hospital discharge suggests this role is not being fulfilled. Indeed, current recommendations,[13] such as for a structured review of needs beyond the first six weeks after discharge, are not being implemented.[14]

We have developed a novel multi-factorial primary care model to address the longer-term needs of stroke survivors living in the community. The components of the model have been assessed for feasibility of delivery within primary care across four general practices prior to starting the IPCAS trial. This led to several minor procedural amendments aimed at improving implementation of the intervention.

Aims

The IPCAS trial (**I**mproving **P**rimary **C**are **A**fter **S**troke) aims to evaluate the clinical and cost effectiveness of a new model of primary care for stroke survivors living in the community compared with standard care.

The primary endpoint for the trial will be two sub-scales (emotion and handicap) of the Stroke Impact Scale (SIS v3.0)[15] as co-primary outcomes at 12 months (adjusted for baseline).

Trial Design

Two-arm cluster randomised controlled trial with general practices as the unit of randomisation.

Randomisation will be performed as random permuted block randomisation with a 1:1 allocation stratified by GP practice size.

Setting and participants

GP practices with a stroke register comprising a minimum of 100 patients and representing a range of urban/rural and different socio-economic status from the East of England and the East Midlands will be recruited. We aim to recruit approximately 920 people with a confirmed history of stroke registered with 46 general practices. Given that primary care addresses the long term needs of stroke survivors we did not restrict participants to any specific time interval after their stroke.

Inclusion Criteria

- On practice register with a history of stroke
- Able to provide written informed consent (with or without the help of a carer)
- Age 18 years or older

Exclusion Criteria

- Patients on the palliative care register
- Living in a nursing home

For peer review only - http://bmjopen.bmj.com/site/about/guidelines.xhtml

Methods

Recruitment of stroke survivors

Prior to practice level randomisation (see below) electronic searches of the clinical computer system will generate a list of people with a history of stroke who meet the inclusion criteria for the study.

Potentially eligible participants will be sent an invitation by their General Practice to take part in the study. If practices had 110 or fewer such people, invitations were sent to all those eligible. For larger practices, a random sample of 110 eligible patients were sent invitations. The invitation pack contains an invitation cover letter, the Patient Information Sheet (PIS), consent form (Appendix 1), a questionnaire containing the co-primary outcomes, and instructions to return the consent form and questionnaire to the researchers in a pre-paid envelope (provided). If no response is received within 2 weeks from the initial mail-out, the practice will send a reminder. If no response is received after the reminder then no further attempts at contact will be made.

Randomisation

Once all invitation letters and reminders have been sent out to patients within a practice, the GP practice will be randomised to intervention or control (ratio of 1:1). Randomisation will be performed centrally by the trial statistician using a stratified, random permuted block design. The stratification factor will be GP practice size, split into two levels: ≤10,500 and >10,500 patients, which reflects the median GP list size in the catchment area. The IPCAS trial flowchart can be seen in figure 1.

Figure 1 about here.

Intervention

The new model of care incorporates a multi-faceted package of service aimed at providing a structured review of stroke care needs, a self-management programme for survivors and their carers, optimised communication between patients and health care services, enhanced communication pathways between the different care services, and increased awareness of and access to national and local community and charity provided services. A logic model depicting the rationale for the IPCAS trial intervention can be seen in figure 2.

Figure 2 about here.

i) Structured Review of Patient Needs:

A structured review will be performed by a practice nurse or other appropriately trained member of the Practice team. Consenting patients will be invited for review by the practice in same the way that they would normally be contacted (e.g. post, telephone or SMS). Where practicable, this review will be incorporated into the regular annual review recommended by current guidelines.[13] A 15-item checklist of common post-stroke needs[16] adapted from a checklist recommended by the WSO[17] will be sent to the stroke survivor in advance, who will be asked to tick all needs which apply to them, and to bring this to the appointment. At the review, the patient will be asked which of the ticked items is their priority for immediate attention. Practice staff will discuss and address up to three key needs prioritised by the patient.

For peer review only - http://bmjopen.bmj.com/site/about/guidelines.xhtml

The review will last approximately 20-30 minutes and may include a routine physical check-up (e.g. blood pressure, record of immunisation, and medication review dependent upon normal clinical practice at the GP surgery) followed by the discussion of post-stroke care needs as identified by the stroke survivor. The outcome of the review will be an action plan agreed with the stroke survivor on how to address each of the key needs identified in the review.

The patient will be provided with an information leaflet introducing the self-management programme, with instructions on how to get further information and how to access the programme.

ii) Self-management Programme (MLAS):
"My Life After Stroke" (MLAS) is a theory-driven self-management education programme with an explicit philosophical underpinning for stroke survivors and their carers (where appropriate) consisting of an initial individual preparatory session, 4 weekly group-based sessions, and a final individual session. Individual appointments last approximately 30-45 minutes. Group sessions will include stroke survivors and their carers (where relevant) and lasts approximately 2½ hours (including breaks).

Group sessions cover a variety of topics including risk factors for stroke and prevention, psychological well-being, information, social needs, problem solving and goal setting. Participants will be given a handbook containing educational content and further information based on the session topics.

The programme will be run by two trained facilitators (health care professionals or people working in the voluntary sector) with an interest in or experience of stroke. All sessions will be held at a suitable, accessible, local community facility.

iii) Direct Point of Contact:
A direct point of contact at the GP surgery will be provided for stroke survivors and their carers. The staff member conducting the enhanced annual review will explain how to access the direct point of contact. Survivors or carers will be able to call the practice and indicate that they would like to talk to someone about a stroke related problem. A single or several Practice nurses or other appropriately trained health care members of the Practice team will assume the role. If none of these people are available at the time of the call, a designated member of the care team will phone back. The aim of the direct point of contact will be to provide support and advice for stroke specific issues, arranging follow-up appointments, and signposting to further specialist or community services.

iv) Enhanced Communication Pathways:
We will arrange a meeting between primary care staff from several practices and specialist staff (hospital and community) to facilitate primary/secondary care communication going forward. All Practice staff involved with the care of stroke survivors will be encouraged to attend additional training/ meetings organised by the specialist services, and given direct contact details for informal communication. Video recordings of local specialist(s) describing their service, the type of patients normally referred to the service, and ways of contacting the service will be made available to all general practice staff.

v) Service Mapping:
To support the information needs regarding local services for stroke related problems, the care team will be provided with a catalogue of stroke (and other relevant) services in participating localities, including information on how to access them. This resource will be available in several electronic and hard copy formats to enable easy access by staff at the practice.

For peer review only - http://bmjopen.bmj.com/site/about/guidelines.xhtml

vi) Training for General Practice Staff:
Training for practice staff involved in structured stroke reviews will include an overview of stroke and stroke related long-term needs, followed by discussion of vignettes based on items from the stroke review checklist. Practice staff will suggest and discuss with the research team the most suitable course of action in each situation tailored to local context.

The list of key health and social services available in the local area will be provided, and practice staff will be familiarised with the service mapping resource that will be made available to them at the practice. The outcomes of the structured review will be recorded on a template in the patient records. We will discuss with the practice how best to embed the direct point of contact role within the current practice operations. To enable ease of attendance the training will be held in the practice and will last approximately two hours.

Control Arm
General practices randomised to the control arm of the trial will continue to deliver usual stroke care. Currently no standard package of long-term care for stroke survivors exists in Primary Care, and therefore we expect "usual care" to vary between Practices. We will capture information on the key elements of care provided by each participating Practice to enable comparison between the two arms of the trial.

Demographic data: age, gender, ethnicity and post code will be collected via postal questionnaire at the time of invitation to the study.

Primary Outcome

The primary endpoint for the trial will be two sub-scales (emotion and handicap) of the Stroke Impact Scale (SIS v3.0)[15] as co-primary outcomes at 12 months (adjusted for baseline) after randomisation of the Practice.

Secondary Outcomes
To be collected at baseline, six and 12 months:

- SIS Short Form[15]
- EuroQol EQ-5D-5L[18]
- ICEpop CAPability measure for Adults (ICECAP-A)[19]
- Time since stroke
- * Co-morbidity, medication use (prescription & "over the counter" (OTC))
- * Southampton Stroke Self-management questionnaire (SSSQ)[20]
- * Health Literacy Questionnaire (HLQ)[21]

* Collected at 12 months follow-up only.

For peer review only

For peer review only - http://bmjopen.bmj.com/site/about/guidelines.xhtml

Data Collection

In this pragmatic, practice level cluster-randomised trial blinding to treatment allocation of the research team or clinical staff involved in delivering the intervention or control condition is not possible. The primary outcome will be captured by postal questionnaires sent to participants. Only in the event of missing data from the primary outcome will participants be contacted by the research team to either encourage them to return their questionnaire or to complete missing items via telephone. Questionnaire data entry onto an electronic spreadsheet will be outsourced to a third-party provider via secure data transfer for blinded data entry. The "coded-allocation" spreadsheet will then be returned to the trial statistician, who will undertake all analyses independent of the rest of the research team.

Baseline: The primary outcome data (emotion and handicap sub-scales of the SIS) will be collected via postal questionnaire at the time of invitation to the study prior to randomisation of the practice. Secondary outcome data (SIS Short Form, EuroQol EQ-5D-5L, and ICEpop CAPability measure for Adults (ICECAP-A)) will be collected by postal questionnaire after receipt of consent. Non-responders to the secondary outcome questionnaire will be followed-up by telephone or the most appropriate method for a participant with aphasia.

Follow-up: at six and 12 months by postal questionnaire. Non-responders/incomplete responders will be followed up by telephone or the most appropriate method for a participant with aphasia.

A review of the general practice notes of consenting participants will be conducted. Data extracted will include number and nature of primary care visits, secondary care inpatient and outpatient visits, investigations, medications and use of social services.

The IPCAS trial SPIRIT flowchart showing scheduled enrolment, interventions and assessments of participants can be seen in figure 3.

Figure 3 about here.

Patient involvement

Patient and members of the public were involved at several stages of the trial, including the design, management, and conduct of the trial. We received input from stroke survivors in the design of the trial materials and management oversight through membership of the trial steering committee. We carefully assessed the burden of the trial interventions on patients. We continue to have patient involvement with the trial through representation on the Steering Committee and the investigators team. We will seek wider patient and public involvement in the interpretation of the trial findings and in development of an appropriate method of dissemination.

Statistical Methods and Analysis

Sample size

With 23 clusters per arm and an average of 20 patients per cluster, assuming an intra-class correlation of 0.03, a typical coefficient of variation of the cluster size of 0.65[22], and 2.5% significance (adjusted to 2.5% because of the use of two co-primary outcomes), we would be able to detect an effect size of 0.33 with at least 90% power on the co-primary outcomes (emotion and handicap sub-scales of the Stroke Impact Scale (SIS v3.0[15])). The sample size calculation has been inflated to allow for a rate of 20% loss to follow-up for patients within clusters. Loss to follow-up of entire clusters is not anticipated.

For peer review only - http://bmjopen.bmj.com/site/about/guidelines.xhtml

Analysis of Primary Outcome
We will use intention to treat (ITT) methods for the analysis of the primary end-points. A mixed effects model will be used to model each of the co-primary outcomes with a cluster random effect and fixed effects for the intervention and covariates that might potentially confound the relationship. Distributional assumptions will be assessed graphically by residual q-q plots and residual by fitted value plots. To handle the co-primary outcomes, 97.5% confidence intervals will be reported for the two primary treatment effects which are equivalent to having the Bonferonni correction on the planned 5% significance level for a single endpoint.

Missing data will be analysed under the assumptions of missing completely at random and missing at random. Multiple imputation will be used to impute missing outcome data and the various potential predictors of missingness will be included in the imputation model.

Secondary analysis will look at the effect of time since stroke on uptake and effectiveness of the intervention.

Economic evaluation
The cost-effectiveness (cost-utility) of the new system of care (intervention package) compared with usual care will be determined in a within-trial economic evaluation. Data will be collected via electronic primary care records and patient questionnaires on resource use implications of the intervention (including training), primary care visits, secondary care inpatient and outpatient visits, investigations, medications and use of social services. Patient and carer-incurred costs will also be considered to allow analysis from a broader societal perspective. Data collection will be undertaken within the trial to determine the time taken to deliver the structured review, and any additional resources required. Attendance at the individual and group MLAS sessions will also be recorded for every participant, and each session will be costed, taking into account staff time, any consumables and use of the venue. Standard unit costs will be applied to health care resource use including NHS reference costs, the BNF for medications and Unit Costs for Health and Social Care (PSSRU).

The main outcomes of interest from the trial are quality of life (measured using EQ 5D-5L[18] at baseline and six and 12 months after entry into the trial) and capability (using the ICECAP-A questionnaire[19]). Initially, a cost-consequence analysis will be performed, to present a disaggregated analysis of all mean resource use and costs related to the intervention and usual care, health care, social care, patient/carer costs and EQ-5D-5L and ICECAP-A scores at all time points. Quality-adjusted life years (QALYs) will be calculated by the area-under-the-curve method using responses at all time points, and adjusted for baseline covariates including EQ-5D-5L score. Multiple imputation will be undertaken where there is missing cost and outcome data. An incremental cost-utility analysis will then be undertaken to determine the cost per QALY gained of the intervention compared with usual care.

To explore uncertainties in the analyses, deterministic sensitivity analysis is proposed to test the robustness of the results when varying key assumptions (for example, length of time required to deliver the intervention).

Process evaluation
A process evaluation will examine the implementation of the IPCAS trial using both quantitative and qualitative methods. As well as capturing process variables, the evaluation will also entail a multidimensional approach to assessing intervention fidelity – the extent to which an intervention is delivered as planned.[23] Using the US National Institutes of Health Behaviour Change Consortium (NIHBCC) guidance[24] we will conduct a 'whole picture' assessment of the intervention across five fidelity dimensions: 1) design, 2) training, 3) delivery, 4) receipt, and 5) enactment. An overview is provided below, with the full protocol reported elsewhere (currently in submission).

For peer review only - http://bmjopen.bmj.com/site/about/guidelines.xhtml

Fidelity of design will be assessed through mapping intervention components to its purported theoretical frameworks. All intervention components have been specified a priori and recorded. Additionally, treatment differentiation (i.e. extent to which intervention and control group practices differ) is considered by comparing the contents of the intervention vs usual care. Fidelity of training will be assessed using self-complete questionnaires (MLAS), video-recorded observations (MLAS) and audio-recorded observations (IPCAS). Fidelity of delivery will be assessed through audio-recorded observations (IPCAS), structured telephone calls to healthcare professionals, and direct observations (MLAS). In addition, semistructured interviews will be conducted with healthcare professionals delivering the intervention, which will help to assess both training and delivery. Fidelity of receipt and enactment will be assessed using self-complete questionnaires (MLAS), structured telephone calls and semistructured interviews with participants.

Analysis

Quantitative aspects of the process evaluation (e.g. process variables, coded video-recorded observations, self-complete questionnaires) will be synthesised descriptively. This will include what factors predict intervention fidelity. Qualitative aspects of the process evaluation (e.g. semistructured interviews, qualitative data from questionnaires) will be synthesised using deductive thematic analysis, using the specific domains from the NIHBCC guidance.

Reporting Adverse Events

We are not anticipating any intervention-related adverse events. Nevertheless, in accordance with Good Clinical Practice (GCP), each Principal Investigator is responsible for reporting all non-exempt SAEs to the Chief Investigator (CI) within 24 hours of first notification. The CI is responsible for ensuring the assessment of all SAEs for expectedness and relatedness is completed and the onward notification of all non-exempt SAEs to the Sponsor within 24 hours of first notification.

Author Statement

The study was conceived and designed by JM and RM. RM and JM drafted the manuscript with contributions from MA (process evaluation, intervention fidelity), SD (statistical analysis), VJ (design/description of MLAS programme), SJ (health economic evaluation) and EK (training of clinical staff, participant recruitment). All authors contributed to the design of the trial, and review of the final manuscript. The IPCAS investigator team supported the design of the trial and provided comments/revisions to the writing of the final manuscript.

Trial Management

The trial is co-sponsored by NHS Cambridgeshire and Peterborough CCG and the University of Cambridge. The study team work with local Clinical Research Networks (CRNs) in the East of England and the East Midlands to identify and recruit GP practices.

Oversight of the trial will fall to an independent committee fulfilling the combined roles of Trial Steering Committee (TSC) and Data Monitoring Committee (DMC). They will provide overall supervision of the conduct of the trial on behalf of the trial sponsor(s) in accordance with NIHR recommendations.[25,26] There are no pre-specified criteria for electively stopping the trial prematurely. In the event that the joint TSC/DMC raise concerns over the safety of participants or the scientific integrity of the trial, a decision as to whether to continue will be discussed and voted upon in keeping with the Terms of Reference of the committees and with Good Clinical Practice in Research guidelines.

Data Management and storage

Data completed by participants, such as consent forms and questionnaires, will be returned to the study team via post using pre-paid stamped addressed envelopes. All relevant data

For peer review only - http://bmjopen.bmj.com/site/about/guidelines.xhtml

collected at practice sites will be sent to the study team by trained and delegated practice staff via a secure transfer server. Paper data will be stored in locked filing cabinets within a security card-protected building at the University of Cambridge. Electronic data (including audio-recordings) will be stored on a Secure Data Hosting Service (SDHS) protected by a dual authentication located on a firewall-protected virtual network (VLAN). Access to study data is restricted to the study team by dual authentication and group permissions. All investigators and trial site staff involved in this trial will comply with the requirements of the General Data Protection Regulation (EU) 2016/679 with regards to the collection, storage, processing and disclosure of personal information.

Declaration of interests: None

References

1. Gulliford MC, Charlton J, Rudd A, et al. Declining 1-year case-fatality of stroke and increasing coverage of vascular risk management: population-based cohort study. Journal of Neurology, Neurosurgery & Psychiatry 2010;81(4):416-22.

2. Lee S, Shafe ACE, Cowie MR. UK stroke incidence, mortality and cardiovascular risk management 1999–2008: time-trend analysis from the General Practice Research Database. BMJ Open 2011;1(2).

3. McKevitt C, Fudge N, Redfern J, et al. Self-reported long-term needs after stroke. Stroke 2011;42(5):1398-403.

4. National Audit Office. Progress in improving stroke care. Department of Health, 2010.

5. Royal College of Physicians CEaEUobotISWP. Sentinel stroke national audit programme (SSNAP): clinical audit April - June 2014. Sentinel Stroke National Audit Programme (SSNAP); 2014.

6. Murray J, Young J, Forster A. Review of longer-term problems after a disabling stroke. Rev Clin Gerontol 2007;17(04):277-92.

7. Logan P, Armstrong S, Avery A, Barer D, Barton G, Darby J, et al. Rehabilitation aimed at improving outdoor mobility for people after stroke: a multi-centre randomised controlled study (the Getting out of the House Study). Health Technol Assess 2014;18(29)

8. Aziz Noor A, Leonardi-Bee J, Phillips Margaret F, Gladman J, Legg Lynn A, Walker M. Therapy-based rehabilitation services for patients living at home more than one year after stroke. Cochrane Database Syst Rev. 2008;(2): [CD005952 p.].

9. Rodgers H, Shaw L, Bhattarai N, Cant R, Drummond A, Ford G, Forster A, Francis R, Hills K, Howel D, Laverty A, McKevitt C, McMeekin P, Price C, Stamp E, Stevens E, Vale L. A trial to evaluate an extended rehabilitation service for stroke patients (EXTRAS): main results. European Stroke Journal 2018; 3(1S): 3–586 DOI: 10.1177/2396987318770127

10. Fryer CE, Luker JA, McDonnell MN, Hillier SL. Self management programmes for quality of life in people with stroke. Cochrane Database of Systematic Reviews 2016, Issue 8. Art. No.:CD010442. DOI: 10.1002/14651858.CD010442.pub2

11. Parke HL, Epiphaniou E, Pearce G, Taylor SJC, Sheikh A, Griffiths CJ, et al. Self-Management Support Interventions for Stroke Survivors: A Systematic Meta-Review. PLoS ONE 2015;10(7): e0131448. doi:10.1371/journal.pone.0131448

For peer review only - http://bmjopen.bmj.com/site/about/guidelines.xhtml

12. Lennon S, McKenna S, Jones F. Self-management programmes for people post stroke: a systematic review. Clinical Rehabilitation 2013;27(10):867–78

13. Intercollegiate Stroke Working Party. National clinical guideline for stroke. London Royal College of Physicians. 2016.

14. Department of Health. National Stroke Strategy. London. Report Number: 284536, 2007.

15. Jenkinson, C., Fitzpatrick, R., Crocker, H. and Peters, M. The Stroke Impact Scale. Validation in a UK setting and development of a SIS short form and SIS index. Stroke 44 pp 2532-2535, 2013.

16. Turner GM, Mullis R, Lim L, Kreit E, Mant J. Using a checklist to facilitate management of long-term care needs after stroke: Insights from focus groups and a feasibility study. BMC Family Practice; 2019;20:2, https://doi.org/10.1186/s12875-018-0894-3

17. Philp I, Brainin M, Walker MF, et al. Development of a poststroke checklist to standardize follow-up care for stroke survivors. *J Stroke Cerebrovasc Dis*. 2013;22(7):e173–80

18. van Reenen M, Janssen B. EQ-5D-5L user guide: basic information on how to use the EQ-5D-5L instrument. Version 2.1. Rotterdam; 2015.

19. Al-Janabi H, N Flynn T, Coast J. Development of a self-report measure of capability wellbeing for adults: the ICECAP-A. Quality of Life Research. 2012;21(1):167-76.

20. Boger, E., Hankins, M., Demain, S., and Latter, S. Development and psychometric evaluation of a new patient-reprted outcome measure for stroke self-management: The Southampton Stroke Self-Management Questionnaire (SSSMQ). Health and Quality of Life Outcomes 2015;13; 165

21. Osborne RH, Batterham R, Elsworth GR, Hawkins M, Buchbinder R. The grounded theory, psychometric development and initial validation of the Health Literacy Questionnaire (HLQ). BMC Public Health. 2013;13:658.

22. Campbell, M.J. and Walters, S.J. How to Design, Analyse and Report Cluster Randomised Trials in Medicine and Health Related Research. John Wiley & Sons. New York. 2014.

23. Peters DH, Adam T, Alonge O, Agyepong IA, Tran N. Republished research: Implementation research: what it is and how to do it. Br J Sports Med 2014;48:731-736.

24. Bellg, A. J., Borrelli, B., Resnick, B., Hecht, J., Minicucci, D. S., Ory, M., et al. Treatment Fidelity Workgroup of the NIH Behavior Change Consortium. Enhancing Treatment Fidelity in Health Behavior Change Studies: Best Practices and Recommendations From the NIH Behavior Change Consortium. Health Psychology 2004;23(5), 443-451. http://dx.doi.org/10.1037/0278-6133.23.5.443

25. NIHR Good Clinical Practice (GCP) Reference Guide (Version 3.1 August 2016) https://www.nihr.ac.uk/our-faculty/documents/GCPReferenceGuide.pdf

For peer review only

For peer review only - http://bmjopen.bmj.com/site/about/guidelines.xhtml

26. NIHR: A Guide to Efficient Trial Management; Trial-Managers'-Network-Guide Fourth Edition: January 2014 ISBN 9780854329748

Legend for Appendix and figures

Appendix 1 IPCAS Trial patient consent form

Figure 1 IPCAS Trial flowchart

Figure 2 Logic model for the IPCAS Trial intervention

Figure 3 IPCAS Trial SPIRIT flowchart showing scheduled enrolment, interventions and assessments of participants.

For peer review only

For peer review only - http://bmjopen.bmj.com/site/about/guidelines.xhtml

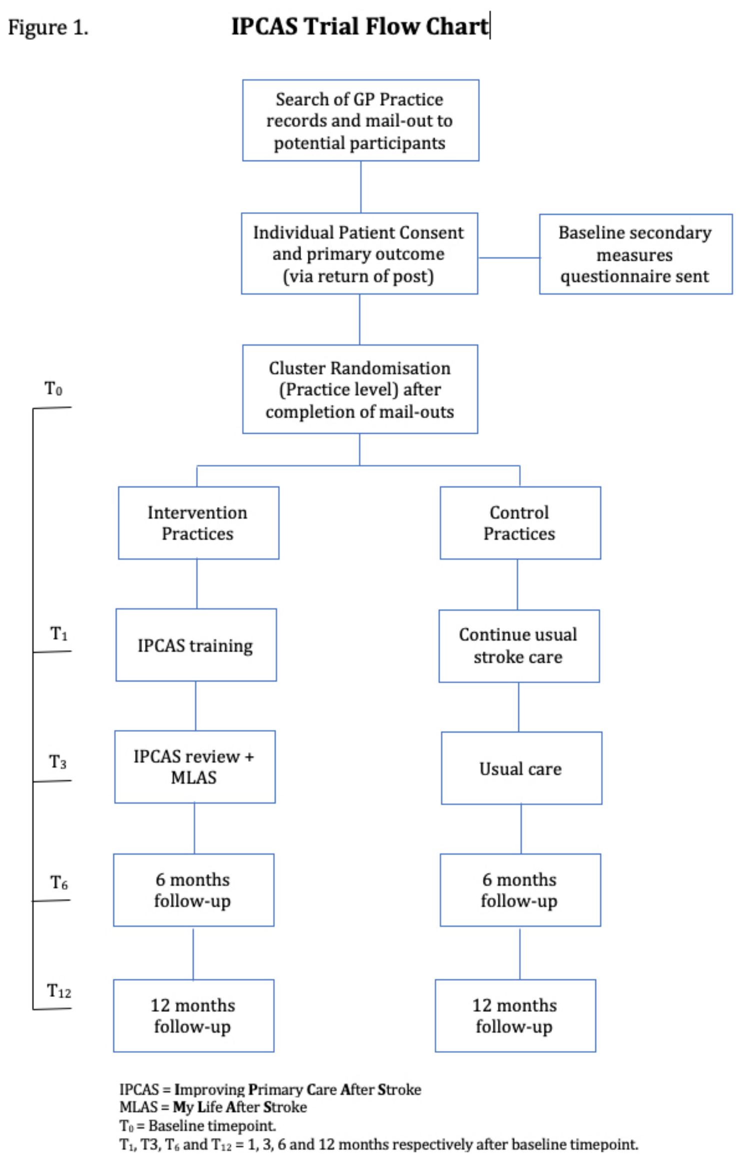

Figure 1 IPCAS Trial flowchart

230x340mm (72 x 72 DPI)

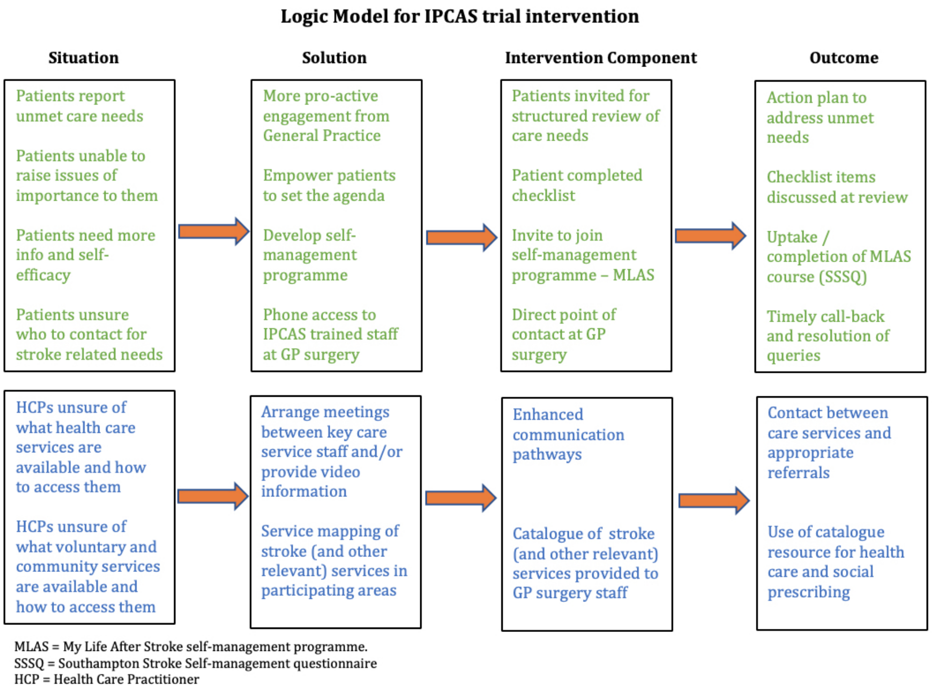

Figure 2 Logic model for the IPCAS Trial intervention

400x288mm (72 x 72 DPI)

Figure 3. Scheduled enrolment, interventions, and assessments of participants.

	STUDY PERIOD			
	**Enrolment**	**Allocation**	**Post-allocation**	
**TIMEPOINT**	***-t_1***	**0**	***6 months***	***12 months***
**ENROLMENT:**				
**Informed consent**	X			
**Allocation**		X		
**INTERVENTIONS:**				
[Intervention]				
[Control]				
**ASSESSMENTS:**				
[Baseline variables]	X			
[Primary outcomes]			X	X
[Secondary outcomes]			X	X

Figure 3 IPCAS Trial SPIRIT flowchart showing scheduled enrolment, interventions and assessments of participants.

319x250mm (72 x 72 DPI)

iPcas improving primary care after stroke

UNIVERSITY OF CAMBRIDGE Public Health and Primary Care The Primary Care Unit

University Hospitals of Leicester NHS NHS Trust

Improving Primary Care After Stroke

TRIAL CONSENT FORM - STROKE SURVIVOR

Version 2.0

Participant ID: *<Participant ID>*

Please **initial** each box

1. I have read and understood the information sheet (*version, dated*) for the above study, and have had the opportunity to ask questions. ☐
2. I understand that I can stop taking part in the research at any time. If I stop I do not have to give a reason. My medical care or legal rights will not be affected. ☐
3. I understand that my GP will be informed of my participation in this study. ☐
4. I understand that the researchers may want to look at relevant sections of my medical notes. I give permission for researchers to have access to my medical records. ☐
5. I understand that my **confidential data** (e.g. my name and address) will be stored securely, and only accessed by the research team. ☐
6. I understand that my **anonymised** data will be archived and may be shared with other researchers and used in future studies. ☐
7. **I agree to take part in this study.** ☐

The following statement is **optional**. Please **only initial the box** if you **agree** to the statement**.**

8. I agree for my annual review to be audiotaped. ☐

Name of Participant: ________________________ **Date:** ________________

Signature: ______________________

Improving primary care after stroke (IPCAS): A cluster randomised controlled trial
Consent Form
IRAS Number: 233891 v 2.0 19-12-17

227x322mm (72 x 72 DPI)

SPIRIT 2013 Checklist: Recommended items to address in a clinical trial protocol and related documents*

Section/item	Item No	Description	Addressed on page number
**Administrative information**			
Title	1	Descriptive title identifying the study design, population, interventions, and, if applicable, trial acronym	______1________
Trial registration	2a	Trial identifier and registry name. If not yet registered, name of intended registry	______2________
	2b	All items from the World Health Organization Trial Registration Data Set	_____1 - 6______
Protocol version	3	Date and version identifier	___Footer_____
Funding	4	Sources and types of financial, material, and other support	______2________
Roles and responsibilities	5a	Names, affiliations, and roles of protocol contributors	______1________
	5b	Name and contact information for the trial sponsor	______2________
	5c	Role of study sponsor and funders, if any, in study design; collection, management, analysis, and interpretation of data; writing of the report; and the decision to submit the report for publication, including whether they will have ultimate authority over any of these activities	______2________
	5d	Composition, roles, and responsibilities of the coordinating centre, steering committee, endpoint adjudication committee, data management team, and other individuals or groups overseeing the trial, if applicable (see Item 21a for data monitoring committee)	______9______

For peer review only

Introduction

Background and rationale	6a	Description of research question and justification for undertaking the trial, including summary of relevant studies (published and unpublished) examining benefits and harms for each intervention	______2________
	6b	Explanation for choice of comparators	______2________
Objectives	7	Specific objectives or hypotheses	______3________
Trial design	8	Description of trial design including type of trial (eg, parallel group, crossover, factorial, single group), allocation ratio, and framework (eg, superiority, equivalence, noninferiority, exploratory)	______3________

Methods: Participants, interventions, and outcomes

Study setting	9	Description of study settings (eg, community clinic, academic hospital) and list of countries where data will be collected. Reference to where list of study sites can be obtained	______3________
Eligibility criteria	10	Inclusion and exclusion criteria for participants. If applicable, eligibility criteria for study centres and individuals who will perform the interventions (eg, surgeons, psychotherapists)	______3________
Interventions	11a	Interventions for each group with sufficient detail to allow replication, including how and when they will be administered	_____4 – 6______
	11b	Criteria for discontinuing or modifying allocated interventions for a given trial participant (eg, drug dose change in response to harms, participant request, or improving/worsening disease)	______N/A______
	11c	Strategies to improve adherence to intervention protocols, and any procedures for monitoring adherence (eg, drug tablet return, laboratory tests)	______4________
	11d	Relevant concomitant care and interventions that are permitted or prohibited during the trial	______N/A______
Outcomes	12	Primary, secondary, and other outcomes, including the specific measurement variable (eg, systolic blood pressure), analysis metric (eg, change from baseline, final value, time to event), method of aggregation (eg, median, proportion), and time point for each outcome. Explanation of the clinical relevance of chosen efficacy and harm outcomes is strongly recommended	______6________
Participant timeline	13	Time schedule of enrolment, interventions (including any run-ins and washouts), assessments, and visits for participants. A schematic diagram is highly recommended (see Figure)	___Figure 2___

For peer review only - http://bmjopen.bmj.com/site/about/guidelines.xhtml

Sample size	14	Estimated number of participants needed to achieve study objectives and how it was determined, including clinical and statistical assumptions supporting any sample size calculations	_____7_______
Recruitment	15	Strategies for achieving adequate participant enrolment to reach target sample size	_____________

Methods: Assignment of interventions (for controlled trials)

Allocation:

Sequence generation	16a	Method of generating the allocation sequence (eg, computer-generated random numbers), and list of any factors for stratification. To reduce predictability of a random sequence, details of any planned restriction (eg, blocking) should be provided in a separate document that is unavailable to those who enrol participants or assign interventions	_____4_______
Allocation concealment mechanism	16b	Mechanism of implementing the allocation sequence (eg, central telephone; sequentially numbered, opaque, sealed envelopes), describing any steps to conceal the sequence until interventions are assigned	_____4_______
Implementation	16c	Who will generate the allocation sequence, who will enrol participants, and who will assign participants to interventions	_____4_______
Blinding (masking)	17a	Who will be blinded after assignment to interventions (eg, trial participants, care providers, outcome assessors, data analysts), and how	_____6_______
	17b	If blinded, circumstances under which unblinding is permissible, and procedure for revealing a participant's allocated intervention during the trial	_____6_______

Methods: Data collection, management, and analysis

Data collection methods	18a	Plans for assessment and collection of outcome, baseline, and other trial data, including any related processes to promote data quality (eg, duplicate measurements, training of assessors) and a description of study instruments (eg, questionnaires, laboratory tests) along with their reliability and validity, if known. Reference to where data collection forms can be found, if not in the protocol	_____6_____
	18b	Plans to promote participant retention and complete follow-up, including list of any outcome data to be collected for participants who discontinue or deviate from intervention protocols	_____6_______

For peer review only - http://bmjopen.bmj.com/site/about/guidelines.xhtml

Data management	19	Plans for data entry, coding, security, and storage, including any related processes to promote data quality (eg, double data entry; range checks for data values). Reference to where details of data management procedures can be found, if not in the protocol	_____9______
Statistical methods	20a	Statistical methods for analysing primary and secondary outcomes. Reference to where other details of the statistical analysis plan can be found, if not in the protocol	_____7 - 8____
	20b	Methods for any additional analyses (eg, subgroup and adjusted analyses)	______7______
	20c	Definition of analysis population relating to protocol non-adherence (eg, as randomised analysis), and any statistical methods to handle missing data (eg, multiple imputation)	______7______
**Methods: Monitoring**			
Data monitoring	21a	Composition of data monitoring committee (DMC); summary of its role and reporting structure; statement of whether it is independent from the sponsor and competing interests; and reference to where further details about its charter can be found, if not in the protocol. Alternatively, an explanation of why a DMC is not needed	______9______
	21b	Description of any interim analyses and stopping guidelines, including who will have access to these interim results and make the final decision to terminate the trial	______9_____
Harms	22	Plans for collecting, assessing, reporting, and managing solicited and spontaneously reported adverse events and other unintended effects of trial interventions or trial conduct	______8______
Auditing	23	Frequency and procedures for auditing trial conduct, if any, and whether the process will be independent from investigators and the sponsor	______9______
**Ethics and dissemination**			
Research ethics approval	24	Plans for seeking research ethics committee/institutional review board (REC/IRB) approval	______2______
Protocol amendments	25	Plans for communicating important protocol modifications (eg, changes to eligibility criteria, outcomes, analyses) to relevant parties (eg, investigators, REC/IRBs, trial participants, trial registries, journals, regulators)	_____________

Consent or assent	26a	Who will obtain informed consent or assent from potential trial participants or authorised surrogates, and how (see Item 32)	_______3_______
	26b	Additional consent provisions for collection and use of participant data and biological specimens in ancillary studies, if applicable	______N/A______
Confidentiality	27	How personal information about potential and enrolled participants will be collected, shared, and maintained in order to protect confidentiality before, during, and after the trial	_______9_______
Declaration of interests	28	Financial and other competing interests for principal investigators for the overall trial and each study site	_______9_______
Access to data	29	Statement of who will have access to the final trial dataset, and disclosure of contractual agreements that limit such access for investigators	_______9_______
Ancillary and post-trial care	30	Provisions, if any, for ancillary and post-trial care, and for compensation to those who suffer harm from trial participation	_______2_______
Dissemination policy	31a	Plans for investigators and sponsor to communicate trial results to participants, healthcare professionals, the public, and other relevant groups (eg, via publication, reporting in results databases, or other data sharing arrangements), including any publication restrictions	_______7_______
	31b	Authorship eligibility guidelines and any intended use of professional writers	______N/A______
	31c	Plans, if any, for granting public access to the full protocol, participant-level dataset, and statistical code	______________
**Appendices**			
Informed consent materials	32	Model consent form and other related documentation given to participants and authorised surrogates	_______1________
Biological specimens	33	Plans for collection, laboratory evaluation, and storage of biological specimens for genetic or molecular analysis in the current trial and for future use in ancillary studies, if applicable	______N/A______

*It is strongly recommended that this checklist be read in conjunction with the SPIRIT 2013 Explanation & Elaboration for important clarification on the items. Amendments to the protocol should be tracked and dated. The SPIRIT checklist is copyrighted by the SPIRIT Group under the Creative Commons "Attribution-NonCommercial-NoDerivs 3.0 Unported" license.

For peer review only - http://bmjopen.bmj.com/site/about/guidelines.xhtml

BMJ Open

The IPCAS Trial (Improving Primary Care After Stroke): protocol of a randomised controlled trial to evaluate a novel model of care for stroke survivors living in the community.

Journal:	*BMJ Open*
Manuscript ID	bmjopen-2019-030285.R2
Article Type:	Protocol
Date Submitted by the Author:	03-Jul-2019
Complete List of Authors:	Mullis, Ricky; University of Cambridge, General Practice & Primary Care Research Centre<br>Aquino, Maria Raisa Jessica (Ryc); University of Cambridge, General Practice & Primary Care Research Centre<br>Dawson, Sarah; University of Cambridge Institute of Public Health, MRC Biostatistics Unit<br>Johnson, Vicki; Univ Hosp Leicester NHS Trust, Leicester Diabetees Centre<br>Jowett, Sue; University of Birmingham, Health Economics Unit<br>Kreit, Elizabeth; University of Cambridge, General Practice & Primary Care Research Centre<br>Mant, Jonathan; University of Cambridge, General Practice and Primary Care Research Unit
**Primary Subject Heading**:	Cardiovascular medicine
Secondary Subject Heading:	General practice / Family practice
Keywords:	Stroke < NEUROLOGY, PRIMARY CARE, MLAS, IPCAS, self management

SCHOLARONE™
Manuscripts

The IPCAS Trial (**I**mproving **P**rimary **C**are **A**fter **S**troke): protocol of a randomised controlled trial to evaluate a novel model of care for stroke survivors living in the community.

*Ricky Mullis[1], Maria Raisa Aquino[1], Sarah Dawson[2], Vicki Johnson[3], Sue Jowett[4], Elizabeth Kreit[1], Jonathan Mant[1].

On behalf of the IPCAS investigator team: Marian Carey, Melanie Davies, Yvonne Doherty, Kamlesh Khunti, Lisa Lim, Bundy Mackintosh, Adrian Mander, Christopher McKevitt, Martin Roland, Stephen Sutton, Marion Walker, Elizabeth Warburton.

1. Primary Care Unit, Department of Public Health and Primary Care, University of Cambridge, UK.
2. MRC Biostatistics Unit, Cambridge Institute of Public Health, University of Cambridge, UK.
3. Complex Intervention Team, Leicester Diabetes Centre, University Hospitals of Leicester NHS Trust, Leicester, UK.
4. Health Economics Unit, Institute of Applied Health Research, University of Birmingham, UK.

* Corresponding author r.mullis@medschl.cam.ac.uk

Key words: Stroke, primary care, self management, IPCAS, MLAS.

Abstract

Introduction
Survival after stroke is improving, leading to increased demand on primary care and community services to meet the long-term care needs of people living with stroke. No formal primary care based holistic model of care with clinical trial evidence exists to support stroke survivors living in the community, and stroke survivors report that many of their needs are not being met. We have developed a multi-factorial primary care model to address these longer-term needs. We aim to evaluate the clinical and cost effectiveness of this new model of primary care for stroke survivors compared with standard care.

Methods and Analysis
IPCAS is a two-arm cluster-randomised controlled trial with general practices as the unit of randomisation. People on the stroke registers of GP practices will be invited to participate. One arm will receive the IPCAS model of care including a structured review using a checklist; a self-management programme; enhanced communication pathways between primary care and specialist services; direct point of contact for patients. The other arm will receive usual care. We aim to recruit 920 people with stroke registered with 46 general practices.

The primary endpoint is two sub-scales (emotion and handicap) of the Stroke Impact Scale (SIS) as co-primary outcomes at 12 months (adjusted for baseline). Secondary outcomes include: SIS Short Form, EuroQol EQ-5D-5L, ICECAP-A, Southampton Stroke Self-management questionnaire (SSSQ), Health Literacy Questionnaire (HLQ); medication use. Cost-effectiveness of the new model will be determined in a within-trial economic evaluation.

Ethics and Dissemination
Favourable ethical opinion was gained from Yorkshire & Humber-Bradford Leeds NHS Research Ethics Committee. Approval to start was given by the Health Research Authority (HRA) prior to recruitment of participants at any NHS site. Data will be presented at national and international conferences and published in peer-reviewed journals.

For peer review only - http://bmjopen.bmj.com/site/about/guidelines.xhtml

Patient and public involvement helped develop the dissemination plan.

Strengths and limitations of this study

Strengths:

- This research is an evaluation of a systematically developed complex intervention.
- The trial is a randomised controlled design with broad inclusion criteria to maximise generalisability.
- Economic evaluation will determine the cost-effectiveness of the intervention.

Limitations:

- Due to the pragmatic nature of this trial only limited blinding of the research team to treatment allocation is possible.
- Exclusion of nursing home residents will restrict the relevance of the findings for this sub-group of stroke survivors.

Study Registration: ClinicalTrials.gov Identifier: NCT03353519 27th November 2017

Favourable ethical opinion for the research was gained on 19th December 2017 from Yorkshire & The Humber - Bradford Leeds NHS Research Ethics Committee. Approval to start was given by the Health Research Authority (HRA) on 21st December 2017, prior to the recruitment of participants commencing at any NHS site.

Patient recruitment started March 2018.

IRAS project ID: 233891 Protocol number: RG71908 REC reference: 17/YH/0441

The IPCAS trial is co-sponsored by the University of Cambridge and NHS Cambridgeshire & Peterborough Clinical Commissioning Group. This research is covered under Cambridge University's Public Liability and Professional Indemnity policy.

This study is funded by the National Institute for Health Research's Programme Grant for Applied Research titled 'Developing primary care services for stroke survivors' reference PTC-RP-PG-0213-20001. The views expressed are those of the authors and not necessarily those of the NHS, the NIHR or the Department of Health. The chief investigator for the study is Professor Jonathan Mant, University of Cambridge email:jm677@medschl.cam.ac.uk

Background and Rationale

Survival after stroke is improving[1,2] leading to increased demand on primary care services to meet the long-term care needs of people with stroke living in the community. Surveys suggest these needs are not being adequately addressed and that many stroke survivors are dissatisfied with care after discharge from hospital.[3,4] Approximately a third of stroke survivors have moderate to severe levels of disability at 6-months.[5] In addition to the many physical consequences of stroke, commonly reported areas of concern include information needs, feelings of abandonment, problems with communication[3] emotional, psychological and social problems, fatigue, and cognitive sequelae including poor memory and concentration.[6]

Little evidence exists as to how best to support long-term stroke survivors[7] especially beyond the first year after stroke,[8] and recent trials of greater specialist input post discharge from hospital have had mixed results.[7,9] No formal primary care based holistic model of care with

For peer review only

For peer review only - http://bmjopen.bmj.com/site/about/guidelines.xhtml

clinical trial evidence exists to support stroke survivors living in the community, and stroke survivors report that many of their needs are not being met. Systematic reviews have demonstrated that self-management after stroke shows promise, but evidence on aspects such as mood and social tasks remain sparse, with wide confidence intervals around effects on outcomes such as quality of life.[10-12]

Primary care could play an important role in the care of people with stroke, including secondary prevention and risk factor management, supporting access to community services, facilitating transfer back to specialist services, and education and provision of information about stroke. However, the feeling of 'abandonment' of people with stroke after hospital discharge suggests this role is not being fulfilled. Indeed, current recommendations,[13] such as for a structured review of needs beyond the first six weeks after discharge, are not being implemented.[14]

We have developed a novel multi-factorial primary care model to address the longer-term needs of stroke survivors living in the community. The components of the model have been assessed for feasibility of delivery within primary care across four general practices prior to starting the IPCAS trial. This led to several minor procedural amendments aimed at improving implementation of the intervention.

Aims

The IPCAS trial (**I**mproving **P**rimary **C**are **A**fter **S**troke) aims to evaluate the clinical and cost effectiveness of a new model of primary care for stroke survivors living in the community compared with standard care.

The primary endpoint for the trial will be two sub-scales (emotion and handicap) of the Stroke Impact Scale (SIS v3.0)[15] as co-primary outcomes at 12 months (adjusted for baseline).

Trial Design

Two-arm cluster randomised controlled trial with general practices as the unit of randomisation.

Randomisation will be performed as random permuted block randomisation with a 1:1 allocation stratified by GP practice size.

Setting and participants

GP practices with a stroke register comprising a minimum of 100 patients and representing a range of urban/rural and different socio-economic status from the East of England and the East Midlands will be recruited. We aim to recruit approximately 920 people with a confirmed history of stroke registered with 46 general practices. Given that primary care addresses the long term needs of stroke survivors we did not restrict participants to any specific time interval after their stroke.

Inclusion Criteria

- On practice register with a history of stroke
- Able to provide written informed consent (with or without the help of a carer)
- Age 18 years or older

For peer review only - http://bmjopen.bmj.com/site/about/guidelines.xhtml

Exclusion Criteria

- Patients on the palliative care register
- Living in a nursing home

Methods

Recruitment of stroke survivors
Prior to practice level randomisation (see below) electronic searches of the clinical computer system will generate a list of people with a history of stroke who meet the inclusion criteria for the study.

Potentially eligible participants will be sent an invitation by their General Practice to take part in the study. If practices had 110 or fewer such people, invitations were sent to all those eligible. For larger practices, a random sample of 110 eligible patients were sent invitations. The invitation pack contains an invitation cover letter, the Patient Information Sheet (PIS), consent form (Appendix 1), a questionnaire containing the co-primary outcomes, and instructions to return the consent form and questionnaire to the researchers in a pre-paid envelope (provided). If no response is received within 2 weeks from the initial mail-out, the practice will send a reminder. If no response is received after the reminder then no further attempts at contact will be made.

Randomisation
Once all invitation letters and reminders have been sent out to patients within a practice, the GP practice will be randomised to intervention or control (ratio of 1:1). Randomisation will be performed centrally by the trial statistician using a stratified, random permuted block design. The stratification factor will be GP practice size, split into two levels: ≤10,500 and >10,500 patients, which reflects the median GP list size in the catchment area. The IPCAS trial flowchart can be seen in figure 1.

Figure 1 about here.

Intervention
The new model of care incorporates a multi-faceted package of service aimed at providing a structured review of stroke care needs, a self-management programme for survivors and their carers, optimised communication between patients and health care services, enhanced communication pathways between the different care services, and increased awareness of and access to national and local community and charity provided services. A logic model depicting the rationale for the IPCAS trial intervention can be seen in figure 2.

Figure 2 about here.

i) Structured Review of Patient Needs:
A structured review will be performed by a practice nurse or other appropriately trained member of the Practice team. Consenting patients will be invited for review by the practice in same the way that they would normally be contacted (e.g. post, telephone or SMS). Where practicable, this review will be incorporated into the regular annual review recommended by current guidelines.[13] A 15-item checklist of common post-stroke needs[16] adapted from a

For peer review only - http://bmjopen.bmj.com/site/about/guidelines.xhtml

checklist recommended by the WSO[17] will be sent to the stroke survivor in advance, who will be asked to tick all needs which apply to them, and to bring this to the appointment. At the review, the patient will be asked which of the ticked items is their priority for immediate attention. Practice staff will discuss and address up to three key needs prioritised by the patient.

The review will last approximately 20-30 minutes and may include a routine physical check-up (e.g. blood pressure, record of immunisation, and medication review dependent upon normal clinical practice at the GP surgery) followed by the discussion of post-stroke care needs as identified by the stroke survivor. The outcome of the review will be an action plan agreed with the stroke survivor on how to address each of the key needs identified in the review.

The patient will be provided with an information leaflet introducing the self-management programme, with instructions on how to get further information and how to access the programme.

ii) Self-management Programme (MLAS):

"My Life After Stroke" (MLAS) is a theory-driven self-management education programme with an explicit philosophical underpinning for stroke survivors and their carers (where appropriate) consisting of an initial individual preparatory session, 4 weekly group-based sessions, and a final individual session. Individual appointments last approximately 30-45 minutes. Group sessions will include stroke survivors and their carers (where relevant) and lasts approximately 2½ hours (including breaks).

Group sessions cover a variety of topics including risk factors for stroke and prevention, psychological well-being, information, social needs, problem solving and goal setting. Participants will be given a handbook containing educational content and further information based on the session topics.

The programme will be run by two trained facilitators (health care professionals or people working in the voluntary sector) with an interest in or experience of stroke. All sessions will be held at a suitable, accessible, local community facility.

iii) Direct Point of Contact:

A direct point of contact at the GP surgery will be provided for stroke survivors and their carers. The staff member conducting the enhanced annual review will explain how to access the direct point of contact. Survivors or carers will be able to call the practice and indicate that they would like to talk to someone about a stroke related problem. A single or several Practice nurses or other appropriately trained health care members of the Practice team will assume the role. If none of these people are available at the time of the call, a designated member of the care team will phone back. The aim of the direct point of contact will be to provide support and advice for stroke specific issues, arranging follow-up appointments, and signposting to further specialist or community services.

iv) Enhanced Communication Pathways:

We will arrange a meeting between primary care staff from several practices and specialist staff (hospital and community) to facilitate primary/secondary care communication going forward. All Practice staff involved with the care of stroke survivors will be encouraged to attend additional training/ meetings organised by the specialist services, and given direct contact details for informal communication. Video recordings of local specialist(s) describing their service, the type of patients normally referred to the service, and ways of contacting the service will be made available to all general practice staff.

For peer review only - http://bmjopen.bmj.com/site/about/guidelines.xhtml

v) Service Mapping:
To support the information needs regarding local services for stroke related problems, the care team will be provided with a catalogue of stroke (and other relevant) services in participating localities, including information on how to access them. This resource will be available in several electronic and hard copy formats to enable easy access by staff at the practice.

vi) Training for General Practice Staff:
Training for practice staff involved in structured stroke reviews will include an overview of stroke and stroke related long-term needs, followed by discussion of vignettes based on items from the stroke review checklist. Practice staff will suggest and discuss with the research team the most suitable course of action in each situation tailored to local context.

The list of key health and social services available in the local area will be provided, and practice staff will be familiarised with the service mapping resource that will be made available to them at the practice. The outcomes of the structured review will be recorded on a template in the patient records. We will discuss with the practice how best to embed the direct point of contact role within the current practice operations. To enable ease of attendance the training will be held in the practice and will last approximately two hours.

Control Arm
General practices randomised to the control arm of the trial will continue to deliver usual stroke care. Currently no standard package of long-term care for stroke survivors exists in Primary Care, and therefore we expect "usual care" to vary between Practices. We will capture information on the key elements of care provided by each participating Practice to enable comparison between the two arms of the trial.

Demographic data: age, gender, ethnicity and post code will be collected via postal questionnaire at the time of invitation to the study.

Primary Outcome

The primary endpoint for the trial will be two sub-scales (emotion and handicap) of the Stroke Impact Scale (SIS v3.0)[15] as co-primary outcomes at 12 months (adjusted for baseline) after randomisation of the Practice.

Secondary Outcomes
To be collected at baseline, six and 12 months:

- SIS Short Form[15]
- EuroQol EQ-5D-5L[18]
- ICEpop CAPability measure for Adults (ICECAP-A)[19]
- Time since stroke
- * Co-morbidity, medication use (prescription & "over the counter" (OTC))
- * Southampton Stroke Self-management questionnaire (SSSQ)[20]
- * Health Literacy Questionnaire (HLQ)[21]

* Collected at 12 months follow-up only.

For peer review only - http://bmjopen.bmj.com/site/about/guidelines.xhtml

Data Collection

In this pragmatic, practice level cluster-randomised trial blinding to treatment allocation of the research team or clinical staff involved in delivering the intervention or control condition is not possible. The primary outcome will be captured by postal questionnaires sent to participants. Only in the event of missing data from the primary outcome will participants be contacted by the research team to either encourage them to return their questionnaire or to complete missing items via telephone. Questionnaire data entry onto an electronic spreadsheet will be outsourced to a third-party provider via secure data transfer for blinded data entry. The "coded-allocation" spreadsheet will then be returned to the trial statistician, who will undertake all analyses independent of the rest of the research team.

Baseline: The primary outcome data (emotion and handicap sub-scales of the SIS) will be collected via postal questionnaire at the time of invitation to the study prior to randomisation of the practice. Secondary outcome data (SIS Short Form, EuroQol EQ-5D-5L, and ICEpop CAPability measure for Adults (ICECAP-A)) will be collected by postal questionnaire after receipt of consent. Non-responders to the secondary outcome questionnaire will be followed-up by telephone or the most appropriate method for a participant with aphasia.

Follow-up: at six and 12 months by postal questionnaire. Non-responders/incomplete responders will be followed up by telephone or the most appropriate method for a participant with aphasia.

A review of the general practice notes of consenting participants will be conducted. Data extracted will include number and nature of primary care visits, secondary care inpatient and outpatient visits, investigations, medications and use of social services.

The IPCAS trial SPIRIT flowchart showing scheduled enrolment, interventions and assessments of participants can be seen in figure 3.

Figure 3 about here.

Patient involvement

Patient and members of the public were involved at several stages of the trial, including the design, management, and conduct of the trial. We received input from stroke survivors in the design of the trial materials and management oversight through membership of the trial steering committee. We carefully assessed the burden of the trial interventions on patients. We continue to have patient involvement with the trial through representation on the Steering Committee and the investigators team. We will seek wider patient and public involvement in the interpretation of the trial findings and in development of an appropriate method of dissemination.

Statistical Methods and Analysis

Sample size

With 23 clusters per arm and an average of 20 patients per cluster, assuming an intra-class correlation of 0.03, a typical coefficient of variation of the cluster size of 0.65[22], and 2.5% significance (adjusted to 2.5% because of the use of two co-primary outcomes), we would be able to detect an effect size of 0.33 with at least 90% power on the co-primary outcomes (emotion and handicap sub-scales of the Stroke Impact Scale (SIS v3.0[15])). The sample size calculation has been inflated to allow for a rate of 20% loss to follow-up for patients within clusters. Loss to follow-up of entire clusters is not anticipated.

For peer review only - http://bmjopen.bmj.com/site/about/guidelines.xhtml

Analysis of Primary Outcome

We will use intention to treat (ITT) methods for the analysis of the primary end-points. A mixed effects model will be used to model each of the co-primary outcomes with a cluster random effect and fixed effects for the intervention and covariates that might potentially confound the relationship. Distributional assumptions will be assessed graphically by residual q-q plots and residual by fitted value plots. To handle the co-primary outcomes, 97.5% confidence intervals will be reported for the two primary treatment effects which are equivalent to having the Bonferonni correction on the planned 5% significance level for a single endpoint.

Missing data will be analysed under the assumptions of missing completely at random and missing at random. Multiple imputation will be used to impute missing outcome data and the various potential predictors of missingness will be included in the imputation model.

Secondary analysis will look at the effect of time since stroke on uptake and effectiveness of the intervention.

Economic evaluation

The cost-effectiveness (cost-utility) of the new system of care (intervention package) compared with usual care will be determined in a within-trial economic evaluation. Data will be collected via electronic primary care records and patient questionnaires on resource use implications of the intervention (including training), primary care visits, secondary care inpatient and outpatient visits, investigations, medications and use of social services. Patient and carer-incurred costs will also be considered to allow analysis from a broader societal perspective. Data collection will be undertaken within the trial to determine the time taken to deliver the structured review, and any additional resources required. Attendance at the individual and group MLAS sessions will also be recorded for every participant, and each session will be costed, taking into account staff time, any consumables and use of the venue. Standard unit costs will be applied to health care resource use including NHS reference costs, the BNF for medications and Unit Costs for Health and Social Care (PSSRU).

The main outcomes of interest from the trial are quality of life (measured using EQ 5D-5L[18] at baseline and six and 12 months after entry into the trial) and capability (using the ICECAP-A questionnaire[19]). Initially, a cost-consequence analysis will be performed, to present a disaggregated analysis of all mean resource use and costs related to the intervention and usual care, health care, social care, patient/carer costs and EQ-5D-5L and ICECAP-A scores at all time points. Quality-adjusted life years (QALYs) will be calculated by the area-under-the-curve method using responses at all time points, and adjusted for baseline covariates including EQ-5D-5L score. Multiple imputation will be undertaken where there is missing cost and outcome data. An incremental cost-utility analysis will then be undertaken to determine the cost per QALY gained of the intervention compared with usual care.

To explore uncertainties in the analyses, deterministic sensitivity analysis is proposed to test the robustness of the results when varying key assumptions (for example, length of time required to deliver the intervention).

Process evaluation

A process evaluation will examine the implementation of the IPCAS trial using both quantitative and qualitative methods. As well as capturing process variables, the evaluation will also entail a multidimensional approach to assessing intervention fidelity – the extent to which an intervention is delivered as planned.[23] Using the US National Institutes of Health Behaviour Change Consortium (NIHBCC) guidance[24] we will conduct a 'whole picture' assessment of the intervention across five fidelity dimensions: 1) design, 2) training, 3) delivery, 4) receipt, and 5) enactment. An overview is provided below, with the full protocol reported elsewhere (currently in submission).

For peer review only - http://bmjopen.bmj.com/site/about/guidelines.xhtml

Fidelity of design will be assessed through mapping intervention components to its purported theoretical frameworks. All intervention components have been specified a priori and recorded. Additionally, treatment differentiation (i.e. extent to which intervention and control group practices differ) is considered by comparing the contents of the intervention vs usual care. Fidelity of training will be assessed using self-complete questionnaires (MLAS), video-recorded observations (MLAS) and audio-recorded observations (IPCAS). Fidelity of delivery will be assessed through audio-recorded observations (IPCAS), structured telephone calls to healthcare professionals, and direct observations (MLAS). In addition, semistructured interviews will be conducted with healthcare professionals delivering the intervention, which will help to assess both training and delivery. Fidelity of receipt and enactment will be assessed using self-complete questionnaires (MLAS), structured telephone calls and semistructured interviews with participants.

Analysis

Quantitative aspects of the process evaluation (e.g. process variables, coded video-recorded observations, self-complete questionnaires) will be synthesised descriptively. This will include what factors predict intervention fidelity. Qualitative aspects of the process evaluation (e.g. semistructured interviews, qualitative data from questionnaires) will be synthesised using deductive thematic analysis, using the specific domains from the NIHBCC guidance.

Reporting Adverse Events

We are not anticipating any intervention-related adverse events. Nevertheless, in accordance with Good Clinical Practice (GCP), each Principal Investigator is responsible for reporting all non-exempt SAEs to the Chief Investigator (CI) within 24 hours of first notification. The CI is responsible for ensuring the assessment of all SAEs for expectedness and relatedness is completed and the onward notification of all non-exempt SAEs to the Sponsor within 24 hours of first notification.

Author Statement

The study was conceived and designed by JM and RM. RM and JM drafted the manuscript with contributions from MA (process evaluation, intervention fidelity), SD (statistical analysis), VJ (design/description of MLAS programme), SJ (health economic evaluation) and EK (training of clinical staff, participant recruitment). All authors contributed to the design of the trial, and review of the final manuscript. The IPCAS investigator team supported the design of the trial and provided comments/revisions to the writing of the final manuscript.

Trial Management

The trial is co-sponsored by NHS Cambridgeshire and Peterborough CCG and the University of Cambridge. The study team work with local Clinical Research Networks (CRNs) in the East of England and the East Midlands to identify and recruit GP practices.

Oversight of the trial will fall to an independent committee fulfilling the combined roles of Trial Steering Committee (TSC) and Data Monitoring Committee (DMC). They will provide overall supervision of the conduct of the trial on behalf of the trial sponsor(s) in accordance with NIHR recommendations.[25,26] There are no pre-specified criteria for electively stopping the trial prematurely. In the event that the joint TSC/DMC raise concerns over the safety of participants or the scientific integrity of the trial, a decision as to whether to continue will be discussed and voted upon in keeping with the Terms of Reference of the committees and with Good Clinical Practice in Research guidelines.

Data Management and storage

Data completed by participants, such as consent forms and questionnaires, will be returned to the study team via post using pre-paid stamped addressed envelopes. All relevant data collected at practice sites will be sent to the study team by trained and delegated practice staff

For peer review only - http://bmjopen.bmj.com/site/about/guidelines.xhtml

via a secure transfer server. Paper data will be stored in locked filing cabinets within a security card-protected building at the University of Cambridge. Electronic data (including audio-recordings) will be stored on a Secure Data Hosting Service (SDHS) protected by a dual authentication located on a firewall-protected virtual network (VLAN). Access to study data is restricted to the study team by dual authentication and group permissions. All investigators and trial site staff involved in this trial will comply with the requirements of the General Data Protection Regulation (EU) 2016/679 with regards to the collection, storage, processing and disclosure of personal information.

Ethics and Dissemination

Favourable ethical opinion for the research was gained on 19th December 2017 from Yorkshire & The Humber - Bradford Leeds NHS Research Ethics Committee. Approval to start was given by the Health Research Authority (HRA) on 21st December 2017, prior to the recruitment of participants at any NHS site.

Patient and public involvement helped develop the dissemination plan. Data will be presented at national and international conferences and published in peer-reviewed journals.

Declaration of interests: None

References

1. Gulliford MC, Charlton J, Rudd A, et al. Declining 1-year case-fatality of stroke and increasing coverage of vascular risk management: population-based cohort study. Journal of Neurology, Neurosurgery & Psychiatry 2010;81(4):416-22.

2. Lee S, Shafe ACE, Cowie MR. UK stroke incidence, mortality and cardiovascular risk management 1999–2008: time-trend analysis from the General Practice Research Database. BMJ Open 2011;1(2).

3. McKevitt C, Fudge N, Redfern J, et al. Self-reported long-term needs after stroke. Stroke 2011;42(5):1398-403.

4. National Audit Office. Progress in improving stroke care. Department of Health, 2010.

5. Royal College of Physicians CEaEUobotISWP. Sentinel stroke national audit programme (SSNAP): clinical audit April - June 2014. Sentinel Stroke National Audit Programme (SSNAP); 2014.

6. Murray J, Young J, Forster A. Review of longer-term problems after a disabling stroke. Rev Clin Gerontol 2007;17(04):277-92.

7. Logan P, Armstrong S, Avery A, Barer D, Barton G, Darby J, et al. Rehabilitation aimed at improving outdoor mobility for people after stroke: a multi-centre randomised controlled study (the Getting out of the House Study). Health Technol Assess 2014;18(29)

8. Aziz Noor A, Leonardi-Bee J, Phillips Margaret F, Gladman J, Legg Lynn A, Walker M. Therapy-based rehabilitation services for patients living at home more than one year after stroke. Cochrane Database Syst Rev. 2008;(2): [CD005952 p.].

9. Rodgers H, Shaw L, Bhattarai N, Cant R, Drummond A, Ford G, Forster A, Francis R, Hills K, Howel D, Laverty A, McKevitt C, McMeekin P, Price C, Stamp E, Stevens E, Vale

For peer review only - http://bmjopen.bmj.com/site/about/guidelines.xhtml

L. A trial to evaluate an extended rehabilitation service for stroke patients (EXTRAS): main results. European Stroke Journal 2018; 3(1S): 3–586 DOI: 10.1177/2396987318770127

10. Fryer CE, Luker JA, McDonnell MN, Hillier SL. Self management programmes for quality of life in people with stroke. Cochrane Database of Systematic Reviews 2016, Issue 8. Art. No.:CD010442. DOI: 10.1002/14651858.CD010442.pub2

11. Parke HL, Epiphaniou E, Pearce G, Taylor SJC, Sheikh A, Griffiths CJ, et al. Self-Management Support Interventions for Stroke Survivors: A Systematic Meta-Review. PLoS ONE 2015;10(7): e0131448. doi:10.1371/journal.pone.0131448

12. Lennon S, McKenna S, Jones F. Self-management programmes for people post stroke: a systematic review. Clinical Rehabilitation 2013;27(10):867–78

13. Intercollegiate Stroke Working Party. National clinical guideline for stroke. London Royal College of Physicians. 2016.

14. Department of Health. National Stroke Strategy. London. Report Number: 284536, 2007.

15. Jenkinson, C., Fitzpatrick, R., Crocker, H. and Peters, M. The Stroke Impact Scale. Validation in a UK setting and development of a SIS short form and SIS index. Stroke 44 pp 2532-2535, 2013.

16. Turner GM, Mullis R, Lim L, Kreit E, Mant J. Using a checklist to facilitate management of long-term care needs after stroke: Insights from focus groups and a feasibility study. BMC Family Practice; 2019;20:2, https://doi.org/10.1186/s12875-018-0894-3

17. Philp I, Brainin M, Walker MF, et al. Development of a poststroke checklist to standardize follow-up care for stroke survivors. *J Stroke Cerebrovasc Dis*. 2013;22(7):e173–80

18. van Reenen M, Janssen B. EQ-5D-5L user guide: basic information on how to use the EQ-5D-5L instrument. Version 2.1. Rotterdam; 2015.

19. Al-Janabi H, N Flynn T, Coast J. Development of a self-report measure of capability wellbeing for adults: the ICECAP-A. Quality of Life Research. 2012;21(1):167-76.

20. Boger, E., Hankins, M., Demain, S., and Latter, S. Development and psychometric evaluation of a new patient-reprted outcome measure for stroke self-management: The Southampton Stroke Self-Management Questionnaire (SSSMQ). Health and Quality of Life Outcomes 2015;13; 165

21. Osborne RH, Batterham R, Elsworth GR, Hawkins M, Buchbinder R. The grounded theory, psychometric development and initial validation of the Health Literacy Questionnaire (HLQ). BMC Public Health. 2013;13:658.

22. Campbell, M.J. and Walters, S.J. How to Design, Analyse and Report Cluster Randomised Trials in Medicine and Health Related Research. John Wiley & Sons. New York. 2014.

23. Peters DH, Adam T, Alonge O, Agyepong IA, Tran N. Republished research: Implementation research: what it is and how to do it. Br J Sports Med 2014;48:731-736.

For peer review only - http://bmjopen.bmj.com/site/about/guidelines.xhtml

24. Bellg, A. J., Borrelli, B., Resnick, B., Hecht, J., Minicucci, D. S., Ory, M., et al. Treatment Fidelity Workgroup of the NIH Behavior Change Consortium. Enhancing Treatment Fidelity in Health Behavior Change Studies: Best Practices and Recommendations From the NIH Behavior Change Consortium. Health Psychology 2004;23(5), 443-451. http://dx.doi.org/10.1037/0278-6133.23.5.443

25. NIHR Good Clinical Practice (GCP) Reference Guide (Version 3.1 August 2016) https://www.nihr.ac.uk/our-faculty/documents/GCPReferenceGuide.pdf

26. NIHR: A Guide to Efficient Trial Management; Trial-Managers'-Network-Guide Fourth Edition: January 2014 ISBN 9780854329748

For peer review only

For peer review only - http://bmjopen.bmj.com/site/about/guidelines.xhtml

Legend for Appendix and figures

Appendix 1 IPCAS Trial patient consent form

Figure 1 IPCAS Trial flowchart

Figure 2 Logic model for the IPCAS Trial intervention

Figure 3 IPCAS Trial SPIRIT flowchart showing scheduled enrolment, interventions and assessments of participants.

For peer review only

For peer review only - http://bmjopen.bmj.com/site/about/guidelines.xhtml

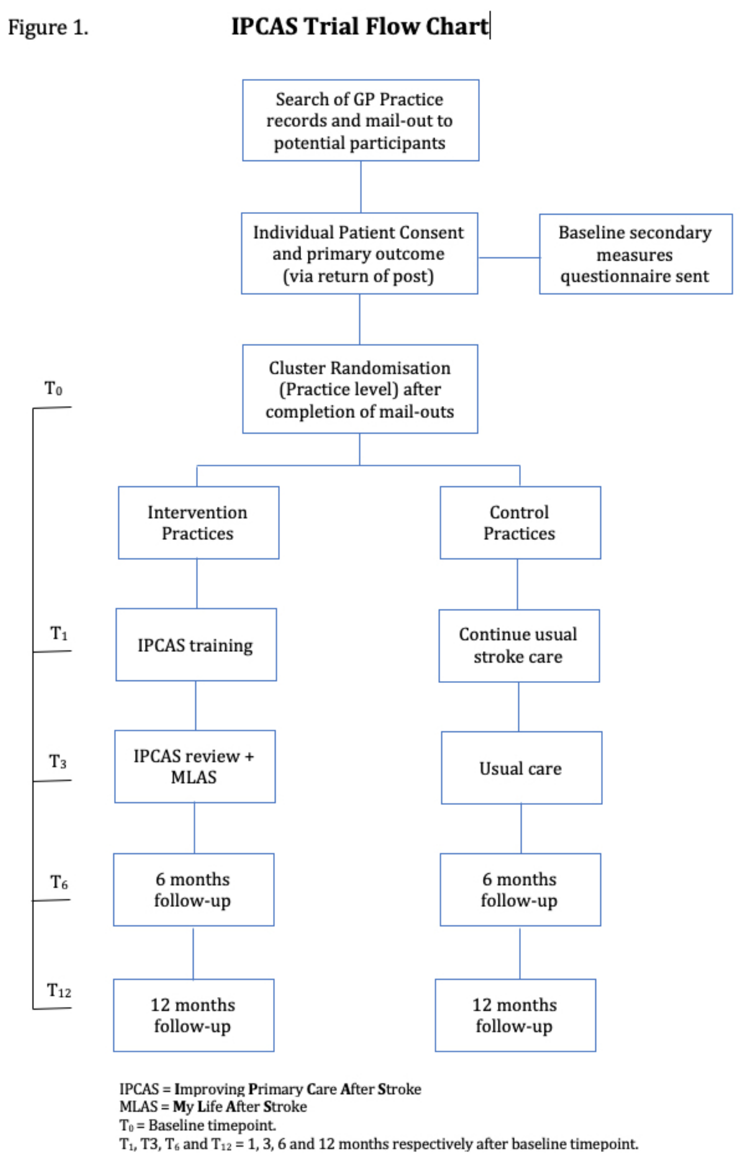

Figure 1 IPCAS Trial flowchart

230x340mm (72 x 72 DPI)

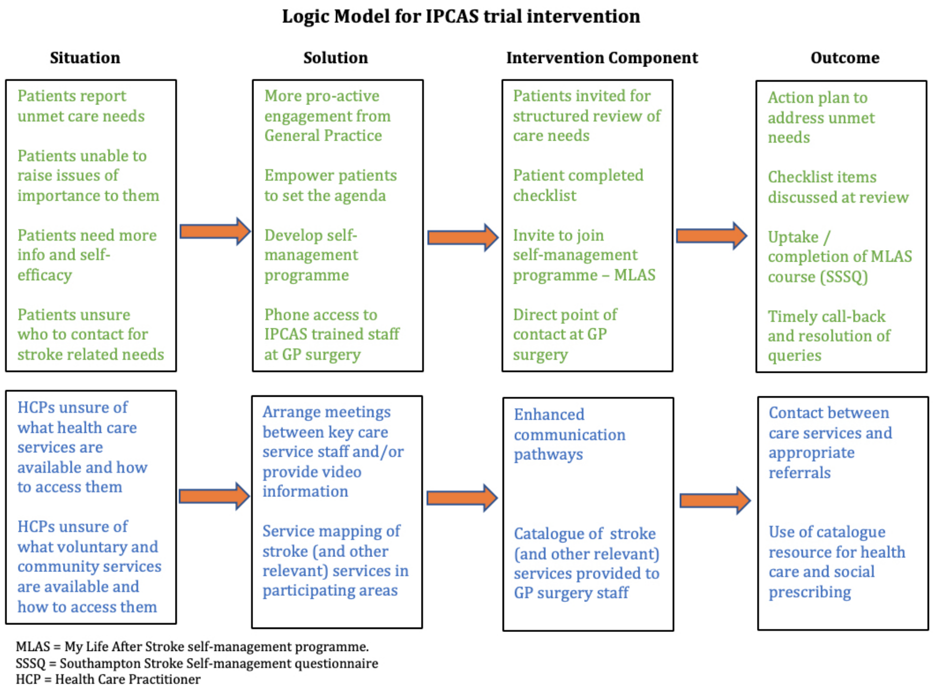

Figure 2 Logic model for the IPCAS Trial intervention

400x288mm (72 x 72 DPI)

Figure 3. Scheduled enrolment, interventions, and assessments of participants.

	STUDY PERIOD			
	Enrolment	Allocation	Post-allocation	
TIMEPOINT	$-t_1$	0	*6 months*	*12 months*
**ENROLMENT:**				
Informed consent	X			
Allocation		X		
**INTERVENTIONS:**				
[Intervention]				
[Control]				
**ASSESSMENTS:**				
[Baseline variables]	X			
[Primary outcomes]			X	X
[Secondary outcomes]			X	X

Figure 3 IPCAS Trial SPIRIT flowchart showing scheduled enrolment, interventions and assessments of participants.

319x250mm (72 x 72 DPI)

iPcas
improving primary care after stroke

UNIVERSITY OF CAMBRIDGE
Public Health and Primary Care
The Primary Care Unit

University Hospitals of Leicester NHS
NHS Trust

Improving Primary Care After Stroke

TRIAL CONSENT FORM - STROKE SURVIVOR

Version 2.0

Participant ID: *<Participant ID>*

Please **initial** each box

1. I have read and understood the information sheet (*version, dated*) for the above study, and have had the opportunity to ask questions. ☐
2. I understand that I can stop taking part in the research at any time. If I stop I do not have to give a reason. My medical care or legal rights will not be affected. ☐
3. I understand that my GP will be informed of my participation in this study. ☐
4. I understand that the researchers may want to look at relevant sections of my medical notes. I give permission for researchers to have access to my medical records. ☐
5. I understand that my **confidential data** (e.g. my name and address) will be stored securely, and only accessed by the research team. ☐
6. I understand that my **anonymised** data will be archived and may be shared with other researchers and used in future studies. ☐
7. **I agree to take part in this study.** ☐

The following statement is **optional**. Please **only initial the box** if you **agree** to the statement.

8. I agree for my annual review to be audiotaped. ☐

Name of Participant: ________________________ **Date:** ________________

Signature: ______________________

Improving primary care after stroke (IPCAS): A cluster randomised controlled trial
Consent Form
IRAS Number: 233891 v 2.0 19-12-17

227x322mm (72 x 72 DPI)

STANDARD PROTOCOL ITEMS: RECOMMENDATIONS FOR INTERVENTIONAL TRIALS

SPIRIT 2013 Checklist: Recommended items to address in a clinical trial protocol and related documents*

Section/item	Item No	Description	Addressed on page number
**Administrative information**			
Title	1	Descriptive title identifying the study design, population, interventions, and, if applicable, trial acronym	_____1_______
Trial registration	2a	Trial identifier and registry name. If not yet registered, name of intended registry	_____2_______
	2b	All items from the World Health Organization Trial Registration Data Set	____1 - 6______
Protocol version	3	Date and version identifier	___Footer_____
Funding	4	Sources and types of financial, material, and other support	_____2_______
Roles and responsibilities	5a	Names, affiliations, and roles of protocol contributors	_____1_______
	5b	Name and contact information for the trial sponsor	_____2_______
	5c	Role of study sponsor and funders, if any, in study design; collection, management, analysis, and interpretation of data; writing of the report; and the decision to submit the report for publication, including whether they will have ultimate authority over any of these activities	_____2_______
	5d	Composition, roles, and responsibilities of the coordinating centre, steering committee, endpoint adjudication committee, data management team, and other individuals or groups overseeing the trial, if applicable (see Item 21a for data monitoring committee)	_____9______

For peer review only

For peer review only - http://bmjopen.bmj.com/site/about/guidelines.xhtml

Introduction

Background and rationale	6a	Description of research question and justification for undertaking the trial, including summary of relevant studies (published and unpublished) examining benefits and harms for each intervention	_____2_______
	6b	Explanation for choice of comparators	_____2_______
Objectives	7	Specific objectives or hypotheses	_____3_______
Trial design	8	Description of trial design including type of trial (eg, parallel group, crossover, factorial, single group), allocation ratio, and framework (eg, superiority, equivalence, noninferiority, exploratory)	_____3_______

Methods: Participants, interventions, and outcomes

Study setting	9	Description of study settings (eg, community clinic, academic hospital) and list of countries where data will be collected. Reference to where list of study sites can be obtained	_____3_______
Eligibility criteria	10	Inclusion and exclusion criteria for participants. If applicable, eligibility criteria for study centres and individuals who will perform the interventions (eg, surgeons, psychotherapists)	_____3_______
Interventions	11a	Interventions for each group with sufficient detail to allow replication, including how and when they will be administered	____4 – 6_____
	11b	Criteria for discontinuing or modifying allocated interventions for a given trial participant (eg, drug dose change in response to harms, participant request, or improving/worsening disease)	_____N/A_____
	11c	Strategies to improve adherence to intervention protocols, and any procedures for monitoring adherence (eg, drug tablet return, laboratory tests)	_____4_______
	11d	Relevant concomitant care and interventions that are permitted or prohibited during the trial	_____N/A_____
Outcomes	12	Primary, secondary, and other outcomes, including the specific measurement variable (eg, systolic blood pressure), analysis metric (eg, change from baseline, final value, time to event), method of aggregation (eg, median, proportion), and time point for each outcome. Explanation of the clinical relevance of chosen efficacy and harm outcomes is strongly recommended	_____6_______
Participant timeline	13	Time schedule of enrolment, interventions (including any run-ins and washouts), assessments, and visits for participants. A schematic diagram is highly recommended (see Figure)	___Figure 2___

For peer review only - http://bmjopen.bmj.com/site/about/guidelines.xhtml

Sample size	14	Estimated number of participants needed to achieve study objectives and how it was determined, including clinical and statistical assumptions supporting any sample size calculations	_____7_______
Recruitment	15	Strategies for achieving adequate participant enrolment to reach target sample size	_____________

Methods: Assignment of interventions (for controlled trials)

Allocation:

Sequence generation	16a	Method of generating the allocation sequence (eg, computer-generated random numbers), and list of any factors for stratification. To reduce predictability of a random sequence, details of any planned restriction (eg, blocking) should be provided in a separate document that is unavailable to those who enrol participants or assign interventions	_____4_______
Allocation concealment mechanism	16b	Mechanism of implementing the allocation sequence (eg, central telephone; sequentially numbered, opaque, sealed envelopes), describing any steps to conceal the sequence until interventions are assigned	_____4_______
Implementation	16c	Who will generate the allocation sequence, who will enrol participants, and who will assign participants to interventions	_____4_______
Blinding (masking)	17a	Who will be blinded after assignment to interventions (eg, trial participants, care providers, outcome assessors, data analysts), and how	_____6_______
	17b	If blinded, circumstances under which unblinding is permissible, and procedure for revealing a participant's allocated intervention during the trial	_____6_______

Methods: Data collection, management, and analysis

Data collection methods	18a	Plans for assessment and collection of outcome, baseline, and other trial data, including any related processes to promote data quality (eg, duplicate measurements, training of assessors) and a description of study instruments (eg, questionnaires, laboratory tests) along with their reliability and validity, if known. Reference to where data collection forms can be found, if not in the protocol	_____6_____
	18b	Plans to promote participant retention and complete follow-up, including list of any outcome data to be collected for participants who discontinue or deviate from intervention protocols	_____6_______

Data management	19	Plans for data entry, coding, security, and storage, including any related processes to promote data quality (eg, double data entry; range checks for data values). Reference to where details of data management procedures can be found, if not in the protocol	_____9______
Statistical methods	20a	Statistical methods for analysing primary and secondary outcomes. Reference to where other details of the statistical analysis plan can be found, if not in the protocol	_____7 - 8____
	20b	Methods for any additional analyses (eg, subgroup and adjusted analyses)	______7______
	20c	Definition of analysis population relating to protocol non-adherence (eg, as randomised analysis), and any statistical methods to handle missing data (eg, multiple imputation)	______7______
**Methods: Monitoring**			
Data monitoring	21a	Composition of data monitoring committee (DMC); summary of its role and reporting structure; statement of whether it is independent from the sponsor and competing interests; and reference to where further details about its charter can be found, if not in the protocol. Alternatively, an explanation of why a DMC is not needed	______9______
	21b	Description of any interim analyses and stopping guidelines, including who will have access to these interim results and make the final decision to terminate the trial	______9_____
Harms	22	Plans for collecting, assessing, reporting, and managing solicited and spontaneously reported adverse events and other unintended effects of trial interventions or trial conduct	______8______
Auditing	23	Frequency and procedures for auditing trial conduct, if any, and whether the process will be independent from investigators and the sponsor	______9______
**Ethics and dissemination**			
Research ethics approval	24	Plans for seeking research ethics committee/institutional review board (REC/IRB) approval	______2______
Protocol amendments	25	Plans for communicating important protocol modifications (eg, changes to eligibility criteria, outcomes, analyses) to relevant parties (eg, investigators, REC/IRBs, trial participants, trial registries, journals, regulators)	_____________

For peer review only - http://bmjopen.bmj.com/site/about/guidelines.xhtml

Consent or assent	26a	Who will obtain informed consent or assent from potential trial participants or authorised surrogates, and how (see Item 32)	3
	26b	Additional consent provisions for collection and use of participant data and biological specimens in ancillary studies, if applicable	N/A
Confidentiality	27	How personal information about potential and enrolled participants will be collected, shared, and maintained in order to protect confidentiality before, during, and after the trial	9
Declaration of interests	28	Financial and other competing interests for principal investigators for the overall trial and each study site	9
Access to data	29	Statement of who will have access to the final trial dataset, and disclosure of contractual agreements that limit such access for investigators	9
Ancillary and post-trial care	30	Provisions, if any, for ancillary and post-trial care, and for compensation to those who suffer harm from trial participation	2
Dissemination policy	31a	Plans for investigators and sponsor to communicate trial results to participants, healthcare professionals, the public, and other relevant groups (eg, via publication, reporting in results databases, or other data sharing arrangements), including any publication restrictions	7
	31b	Authorship eligibility guidelines and any intended use of professional writers	N/A
	31c	Plans, if any, for granting public access to the full protocol, participant-level dataset, and statistical code	
**Appendices**			
Informed consent materials	32	Model consent form and other related documentation given to participants and authorised surrogates	1
Biological specimens	33	Plans for collection, laboratory evaluation, and storage of biological specimens for genetic or molecular analysis in the current trial and for future use in ancillary studies, if applicable	N/A

*It is strongly recommended that this checklist be read in conjunction with the SPIRIT 2013 Explanation & Elaboration for important clarification on the items. Amendments to the protocol should be tracked and dated. The SPIRIT checklist is copyrighted by the SPIRIT Group under the Creative Commons "Attribution-NonCommercial-NoDerivs 3.0 Unported" license.
